# Photoinduced femtosecond spin-flip assisted by a single-mode linear phonon

**DOI:** 10.1126/sciadv.adv9616

**Published:** 2026-02-04

**Authors:** Na Wu, En Wang, Daqiang Chen, Chao Lian, Yaxian Wang, Sheng Meng

**Affiliations:** ^1^ Beijing National Laboratory for Condensed Matter Physics and Institute of Physics, Chinese Academy of Sciences, Beijing 100190, China.; ^2^ School of Physical Sciences, University of Chinese Academy of Sciences, Beijing 100190, China.; ^3^ Songshan Lake Materials Laboratory, Dongguan, Guangdong 523808, China.

## Abstract

Optical manipulation of spin-flip on a picosecond to femtosecond timescale has long been pursued to innovate next-generation electronic devices. However, understanding the ultrafast spin-electron-lattice coupled dynamics remains challenging, especially when the system is driven far from equilibrium. Here, we demonstrate an ultrafast light-induced spin-flip within 300 fs in Fe_3_GeTe_2_, a prototypical two-dimensional itinerant ferromagnet. Notably, by varying the laser fluence, we identify three distinct regimes that emerge assisted by a photoinduced single linear phonon mode, namely demagnetization, spin-flip, and spin-melting. We resolve the dominant role of displacively excited A_1g_ phonons, while nonequilibrium electron occupation is essential to break the degeneracy of the spin up and down states and lower the spin-flip energy barrier. Accompanying the spin-flip, we also observe a sign change of the Berry curvature, implying involvement of nontrivial band topology. Our results provide a general guidance for optical manipulation of spin orders, holding promises for advancing future spintronics and information technology.

## Teaser

Light-driven 300-fs spin-flip in Fe_3_GeTe_2_, via A_1g_ phonons and nonequilibrium electrons, enables ultrafast spintronic control.

## INTRODUCTION

Electron spin, a fundamental degree of freedom in solids, can be encoded with data and used in modern spintronics devices. Although endowed with the promises to outperform traditional electronics with advantages in terms of smaller scale, faster operating speed and less energy dissipation (*1*, *2*), one of the grand challenges lies in the ability to manipulate electron spins and their ensembles in an ultrafast and coherent manner (*3*). Optical manipulation by an ultrashort laser pulse offers a previously unidentified route to deterministically flip spins within the duration of the laser pulse (i.e., picosecond to femtosecond timescale). This surpasses the best that can be expected from overcoming the spin exchange interaction barrier by an electrical or magnetic field. Ever since the observation of femtosecond demagnetization in nickel (*4*), the past decades have witnessed attempts to achieve all-optical manipulation of spin order in ferromagnetic (semi)metals (*5*, *6*), antiferromagnetic alloys (*7*, *8*), thin films and superstructures (*9*), and recently emerging two-dimensional (2D) materials such as CrI_3_ (*10*), Fe_3_GeTe_2_ (*11*, *12*), NiPS_3_ (*13*, *14*), and their bulk counterparts and heterostructures. Theoretical understanding of ultrafast spin dynamics has been advancing in the meantime, with topics focusing on hot carrier dynamics and energy/angular momentum transfer during photoexcitation (*15*, *16*) using mainly phenomenological two-temperature models and their generalization (*4*, *17*). Various microscopic mechanisms have been proposed in literature, including spin-orbit coupling (SOC)–induced spin dynamics (*18*, *19*), intersite spin transfer (*20*, *21*), and phonon-mediated spin-order switch (*13*, *22*–*25*). There, the optically driven phonon modes either act as a quasistatic strain, modifying the potential energy surface (PES) and thus the exchange interaction, or carry and transfer angular momentum when a circularly polarized laser pulse excites circular atomic motion.

Nevertheless, to further reduce the critical transition time of spin-flip, one needs to fully understand the microscopic mechanism, where hurdles lie from multiple aspects. When spin dynamics occurs on a subpicosecond timescale, traditional characterization methods such as Hall conductance (*26*, *27*) and optical imaging (*28*, *29*) do not have the temporal resolution required to capture the spin-flip timescale, while terahertz emission spectroscopy (*30*) and time-resolved (tr) pump probe–based methods including tr-XMCD (x-ray magnetic circular dichroism) and tr-MOKE (magneto-optical Kerr effect) usually convolve the thermal effects and rely on limited but ever-increasing theoretical modeling (*28*). Up to now, time-dependent calculations have primarily focused on the changes in the occupation of excited carriers in different orbitals (*19*, *31*), providing insight into microscopic processes such as SOC and spin exchange effect. The coupled nonequilibrium spin-electron-lattice dynamics, as we will show hereafter to be ubiquitous and important in the ultrafast spin dynamics, has rarely been touched. Therefore, a theoretical description that can capture the electron and lattice excitation and treat their ultrafast dynamics on an equal footing is urgently needed to clarify how they jointly interact with spins, especially when the system is driven far from equilibrium.

**Fig. 1.**
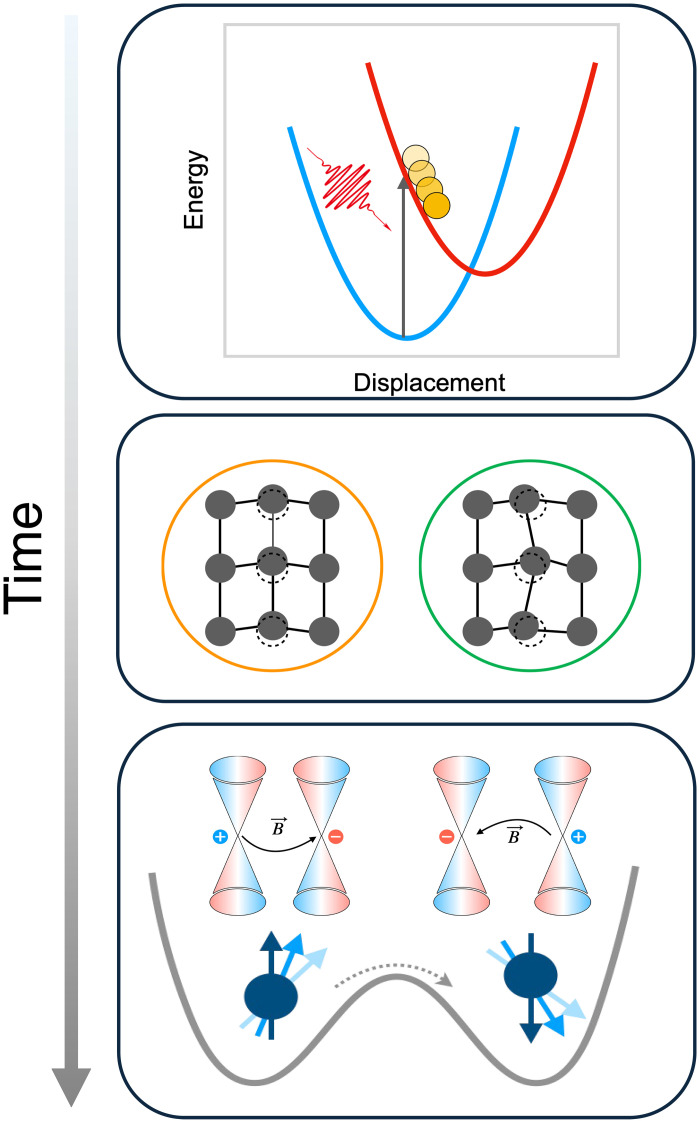
Schematics for the phonon-induced ultrafast spin-flip in monolayer FGT. First, the photoexcited carrier drives a displacive excitation of coherent phonons via electron-phonon coupling. The combination with the lowered energy barrier and nonequilibrium electron distribution leads to a femtosecond spin-flip, accompanying a topology switch in its band structure.

In this work, we investigate light-induced ultrafast spin-flip in the monolayer Fe_3_GeTe_2_ (abbreviated as FGT hereafter), using the real-time time-dependent density functional theory (rt-TDDFT) in combination with Ehrenfest molecular dynamics (*32*–*34*), which allows us to capture the photoinduced spin dynamics and decouple the electronic and structural contributions. Specifically, we observe a remarkably fast reversal of the magnetic moment within 300 fs upon exposure to an intense linearly polarized near-infrared laser pulse. By varying the pump fluence, we construct a phase diagram for optically realizing demagnetization, spin-flip, and spin-melting, respectively, where the spin-flip critical time can vary from 250 to 600 fs. By tracking the real-time lattice evolution, we reveal that out-of-plane (OP) A_1g_ and A_2u_ phonons are coherently excited, the former of which plays a critical role for the spin-flip to occur. By evaluating Berry curvature of the transient band structure, we find that the spin-flip is directly associated with a sign reversal of the Chern number, with a schematic for the chronological light-induced processes shown in Fig. 1. In light of these results, we discuss the necessity of the nonequilibrium electron-phonon interactions for the femtosecond spin-flip in FGT, namely, the displacive excitation of A_1g_ phonon will reduce the energy barrier between the opposite spin polarization, and the asymmetric electronic excitation breaks the degeneracy of spin up and down states. Our work thus proposes a general protocol for light-induced ultrafast spin-flip, thus opening up avenues for advancing information processing and quantum technologies toward an unprecedented ultrafast timescale.

## RESULTS

### Femtosecond spin-flip

**Fig. 2.**
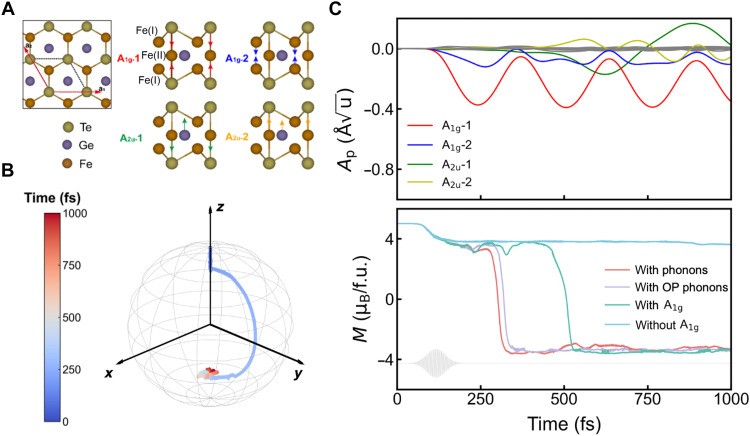
The structural and magnetization (*M_z_*) dynamics of monolayer Fe_3_GeTe_2_ upon optical excitation. (**A**) Top view of the Fe_3_GeTe_2_ crystal structure, along with the side view of the eigenvectors of four zone-center optical phonon modes. (**B**) The ultrafast spin-flip dynamics within 1 ps, represented by an effective spin vector plotted in the spherical coordinate system. Color coding denotes our simulation time. (**C**) Top: The projected phonon amplitudes (*A*_p_) over time, with four large-amplitude *z*-polarized phonon modes highlighted to illustrate their coherent motion. Bottom: The transient dynamics of *z* component magnetic moment (*M_z_*) under different scenarios: with all phonons (in red), with OP phonons (in purple), with A_1g_ phonons only (in green), and without A_1g_ phonons (in cyan). The profile of the applied pump laser is depicted by the gray line.

We use a prototypical 2D itinerant ferromagnet FGT, which has been shown to exhibit strong spin-lattice coupling as manifested in pressure-dependent magnetic configurations (*35*) and anomalous phonon linewidths (*36*). Meanwhile, it exhibits nontrivial band topology (*37*), strong electron correlation (*38*), and tunable Curie temperature (*39*). Bulk FGT crystallizes in a honeycomb lattice and belongs to the *P*6_3_/*mmc* space group (no. 194). The monolayer consists of a five–atomic layer sandwich structure with a mirror plane. The top layers are exclusively occupied by Te atoms, whereas the second layers host the Fe(I) atom. The middle layer consists of both Ge and Fe(II) atoms, as depicted in Fig. 2A. The lattice constant is optimized to be 3.991 Å, with a net magnetic moment of ~5.02 μ_B_/f.u. (f.u. denotes formula unit), consistent with previous studies (for computational details, see Materials and Methods) (*40*, *41*).

We then apply an intense optical excitation using a Gaussian-enveloped, linearly polarized laser with a photon energy of 1.55 eV and a pump fluence of 7.72 mJ/cm^2^. To decouple the electron, spin, and lattice degrees of freedom, we explicitly track time-dependent magnetization in the presence of different lattice dynamics. Technically, the lattice degree of freedom can be manipulated by either relaxing or fixing the atomic positions along different coordinates. The *z* component of magnetization *M_z_*(*t*) is demonstrated in the bottom panel of Fig. 2C, the evolution of which reveals distinct features. In the presence of full lattice degree of freedom, there occurs a spin-flip on an ultrafast timescale of ~250 fs (red curve), on top of the ~20% demagnetization due to the SOC effect. In this case, the light-induced variation of the magnitude and direction of the magnetic moment, corresponding to the longitudinal and transverse relaxation of the spin, can be more clearly observed in the temporal trace of the effective spin vector in Fig. 2B, and the evolution of the *x*/*y* components is shown in note S1.

While previous work mostly treats the lattice as a thermal bath with a characteristic temperature, under strong laser pump, the lattice can be driven coherently where a semiclassical or perturbative description fails. To this end, we project the light-induced transient atomic positions onto phonon eigenvectors, as illustrated in Fig. 2C (top panel). We observe coherent excitation of four optical phonon modes, of which the eigenvectors are shown in Fig. 2A. These four phonon modes can be categorized into A_1g_ and A_2u_ based on their symmetry, with two A_1g_ phonons reserving the mirror symmetry, and two A_2u_ phonons breaking the mirror symmetry. We note that we use the irreducible representations of the bulk material to denote the monolayer, while providing a detailed symmetry analysis in note S10, without loss of generality. The excited large-amplitude A_1g_ modes have also been proven to feature strong coupling to the spin order (*11*). Switching off the A_1g_ phonons eliminates the spin-flip after the demagnetization, with a stabilized *M_z_* value (cyan curve in Fig. 2C). Comparing the magnetization dynamics with the presence of all phonon modes (red curve), only the OP phonons (purple curve), and only A_1g_ modes (green curve) in Fig. 2C, it is evident that A_1g_ modes play a decisive role in the ultrafast spin-flip, while the involvement of other phonons can notably affect the spin-flip critical time (for further discussion see note S4). We note that the symmetry-breaking A_2u_ phonons vibrate around their equilibrium position, although with a large amplitude. Therefore, the A_2u_ phonons are likely not excited from the displacive mechanism, for which our symmetry analysis strongly indicates that the substantial inplane magnetization during the spin-flip imposes a major driving force for the A_2u_ modes (for details, see notes S10 and S14). It is also likely from our analysis on the magnon excitation that the timing of an excitation of the optical magnon branch coincides with the excitation of the A_2u_ modes (note S13). Nevertheless, because our model encodes only the phase angle of the three Fe atoms instead of the spin vector, further study is still needed to draw concrete conclusions.

### Manipulating ultrafast spin dynamics

**Fig. 3.**
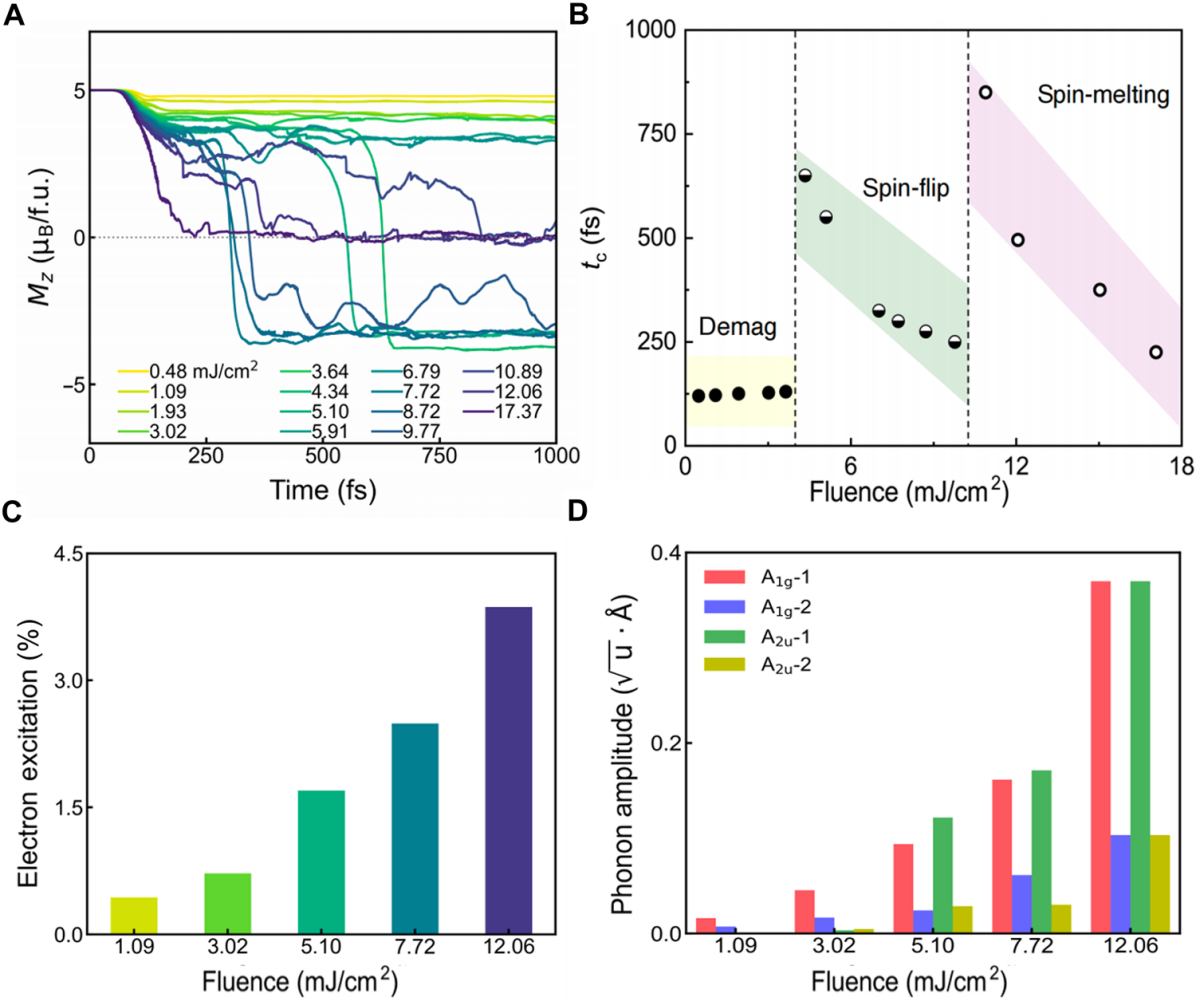
Optical manipulation of the ultrafast spin-flip. (**A**) The temporal evolution of *M_z_* in FGT under different pump fluences ranging from 0.48 to 17.37 mJ/cm^2^. (**B**) Phase diagram of the spin order highlighting regimes of demagnetization (yellow with solid circles), spin-flip (green with half-filled circles), and ferromagnetic spin-melting (pink with open circles), with different transition critical time *t*_c_ and used pump fluence. The *t*_c_ for spin-flip and spin-melting is highly tunable upon increasing the pump fluence. (**C**) The electron excitation and (**D**) amplitudes of optically induced coherent phonon modes with increasing pump fluence.

Having demonstrated the light-induced femtosecond spin-flip, we now seek a general route of optical manipulation of spin order. Figure 3A illustrates the photoinduced magnetization dynamics with laser fluence varying from 0.48 to 17.37 mJ/cm^2^. There emerge three distinct regimes based on the near-equilibrium final magnetic configurations within a picosecond, namely, demagnetization with no sign change for *M_z_*, spin-flip where an after-pulse sign change occurs for *M_z_*, and ferromagnetic spin-melting where the total magnetic moment approaches zero. The characteristic critical transition time, denoted *t*_c_, in each regime is highly tunable, for which we construct the all-optical control of the magnetic phase diagram as shown in Fig. 3B. For demagnetization, *t*_c_ represents the time needed to reach the maximum variation of Δ*M_z_*, i.e., $M_{z}\left(t\right)-M_{z}\left(t=0\right)$. Similarly, the critical time *t*_c_ for spin-flip and ferromagnetic spin-melting can be defined as the time at which *M_z_* switches sign and *M_z_* approaches zero, respectively.

In the weak excitation regime with laser fluence ranging from 0.48 to 3.64 mJ/cm^2^, a substantial reduction of *M_z_* up to 20% appears with *t*_c_ ~ 100 fs (for dynamics of the *x*/*y* components, see note S2), approximately the duration of the laser pulse and independent of laser fluence. Entering the intense excitation regime with fluence ranging from 4.34 to 9.77 mJ/cm^2^, a femtosecond reversal of *M_z_* during after-pulse dynamics becomes evident. The critical time *t*_c_ for the spin-flip is largely tunable by the incident light fluence, with larger fluence resulting in a shorter critical time, i.e., faster spin-flip. In our simulations, the spin-flip *t*_c_ ranging from 250 to 600 fs is achieved before the fluence passes the threshold for spin-melting. When exposed to strong excitation with pump fluence over 10.89 mJ/cm^2^, there is no more macroscopic spin order and the local magnetic moment becomes negligible, resembling a melting behavior (note S3). This is attributed to heat accumulation in the electronic subsystem under intense laser. Similar to the spin-flip regime, the spin-melting critical time also varies with laser fluence, with higher fluence resulting in shorter *t*_c_. As the photoexcited lattice distortion can have a lifetime on the scale of picosecond and the excited electrons a few hundred femtoseconds [1.3 ps and 430 fs, respectively for FGT; (*42*)], the ultrafast magnetization transition can be realized in practice. Furthermore, given that the spin-flip happens within 300 fs, it is possible that fast spin dynamics will experience a phonon bottleneck scenario, and a pump probe–based experiment (e.g., tr-MOKE) could be able to clarify the lifetime for both spins and activated coherent phonons.

The established phase diagram of laser fluence, stabilized spin orders, and their critical time can serve as a practical guiding map for optical manipulation of ultrafast spin-flip in FGT, potentially sparking future experiments. These distinct regimes highlight the nonthermal pathways upon optical excitation, where quasithermodynamic descriptions based on effective temperatures are either impossible or inadequate (*43*).

### Temporal evolution of Berry curvature

To gain a better understanding of the microscopic mechanism for the distinct spin dynamics, we analyze the electronic and phononic excitation, shown in Fig. 3 (C and D). Here, the electron excitation is estimated by the sum of population change after photoexcitation, i.e., $\Delta N=\sum_{k}|f_{k}\left(t\right)-f_{k}\left(0\right)|$, where $f_{k}\left(t\right)=\sum_{n}\sum_{n^{\prime }}^{\mathrm{CB}}{|\langle \psi_{n^{\prime },k}\left(0\right)|\psi_{n,k}\left(t\right)\rangle |}^{2}$ with *n* summing over the band indices and $n^{\prime }$ running over all conduction bands. It might be unexpected at first glimpse that both the excited carriers and the A_1g_ phonon amplitudes exhibit a linear (under weak excitation) or quadratic (under strong excitation) dependence on the laser fluence. However, the spin dynamics fall into distinct regimes as the laser fluence increases. Furthermore, although the magnetization dynamics under different lattice excitations reveal that the ultrafast spin-flip is primarily driven by the displacive excitation of A_1g_ phonon modes, we observe no spin-flip in the absence of the nonequilibrium electron distribution (note S5).

These observations motivate us to examine the temporal evolution of the topological invariants (*44*). Generally speaking, the Chern number, summation of the Berry curvature of occupied bands over the Brillouin zone, can describe the global topological properties and is generally robust against local variations in the electronic structure. It has been well accepted that a nonzero Chern number can result in the macroscopic transport observable, i.e., the anomalous Hall conductivity (*44*, *45*). Meanwhile, the Berry curvature can be regarded as an effective magnetic field in the momentum space, contributing to an anomalous velocity term (*44*, *46*). Therefore, the Chern number can provide a unique view of the nonequilibrium electronic structure, encoding the evolution of eigenstates as a global function in the parameter space. Given that our macroscopic observable is the spin-flip, we thus focus on evaluating the sign change in its Chern number, which is typically associated with a time-reversal operation or an applied magnetic field.

**Fig. 4.**
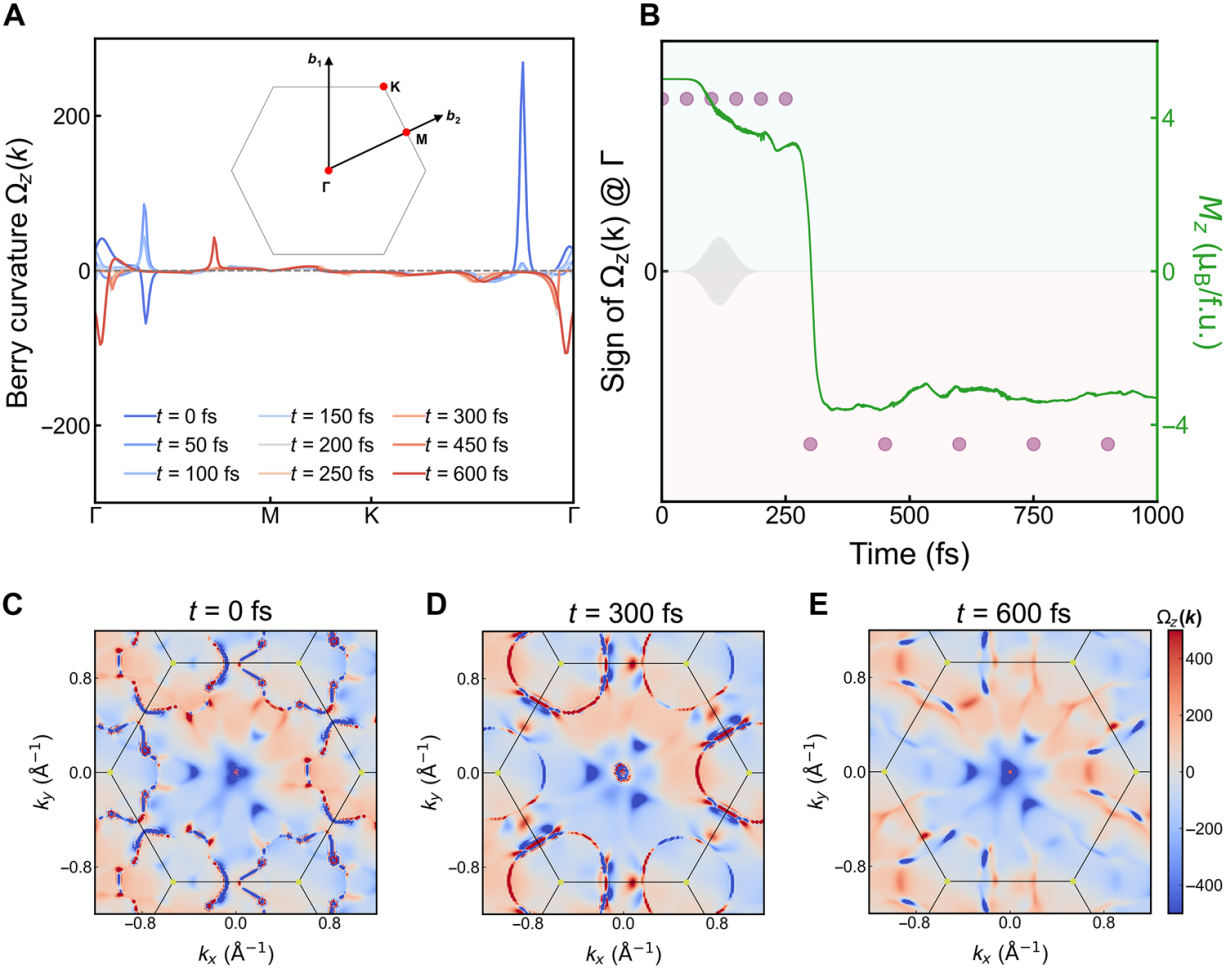
Transient Berry curvature during the spin-flip. (**A**) The *z*-direction Berry curvature $\Omega_{z}\left(k\right)$ along the high-symmetry path Γ-M-K-Γ in the 2D Brillouin zone, calculated at various snapshots under a laser fluence of 7.72 mJ/cm^2^. The inset shows the 2D Brillouin zone and the high-symmetry path. (**B**) The sign flip of the Chern number together with the time-dependent dynamics of the *z*-direction magnetic moment (*M_z_*). (**C** to **E**) Evolution of the Berry curvature distribution in the Brillouin zone for *t* = 0, 300, and 600 fs.

The band structure of FGT exhibits multiple nontrivial gap openings (notes S6 and S7), for which Fig. 4A illustrates its Berry curvature evolution at various moments. Each state evolves independently and undergoes gap closing and opening (note S12), and a shift of the band crossings, as well as a switch of their chirality before and after the spin-flip (*t* > 250 fs) can be seen. Moreover, Fig. 4 (C to E) shows the distribution of Berry curvature in the entire Brillouin zone, where *t* = 0, 300, and 600 fs are chosen to represent the initial (ground) state, the right after spin-flip state, and state after stabilization of the magnetic configuration. Both the distribution of Berry curvature and the sign of Chern number, sgn(C), show abrupt change accompanying the spin-flip, which becomes more evident when compared to situations where no spin-flip occurs, as detailed in note S8. Last, we extract the sign of the Chern number and plot its temporal evolution together with the magnetization dynamics, shown in Fig. 4B. Although we do not see a clear oscillation behavior of its value (note S12), we do observe a definitive accordance between the reversal of sgn(C) and the spin-flip.

### The nonequilibrium PES

To pinpoint the mechanism of the femtosecond spin-flip, we formulate a phenomenological Landau-Ginzburg free-energy model to describe the spin-flip process and the spin-phonon coupling. The order parameters of FGT are the magnetization M and the amplitude of phonon normal modes $Q_{i}$. The noninteracting free energy of FGT can be written as (*47*, *48*)

$$F_{0}=\alpha M^{4}+\beta_{1}M_{z}^{2}+\beta_{2}M_{∥}^{2}+\sum_{i}\frac{1}{2}k_{i}Q_{i}^{2}+\sum_{i}\frac{1}{4}g_{i}Q_{i}^{4}$$ (1)

where M=∣M∣, $\alpha ,\beta_{1,2}$, and $k_{i},g_{i}$ are material parameters. $M_{z}$ and $M_{∥}$ denote the magnetization along and perpendicular to the easy axis, i.e., *z* axis, respectively. We assume $\beta_{1}\neq \beta_{2}$ to describe the magnetic anisotropy. Here, we choose the coordinate system such that the z axis is perpendicular to the plane of FGT and set $|\beta_{1}|>|\beta_{2}|$ to ensure that the system has a local minimum at $M_{z}=\pm M_{0}$ and $M_{∥}=0$, where $M_{0}=\sqrt{\frac{-\beta_{1}}{2\alpha }}$. The energy barrier between the two minima is given by

$$E_{b}=\frac{\beta_{1}^{2}-\beta_{2}^{2}}{4\alpha }$$ (2)

**Fig. 5.**
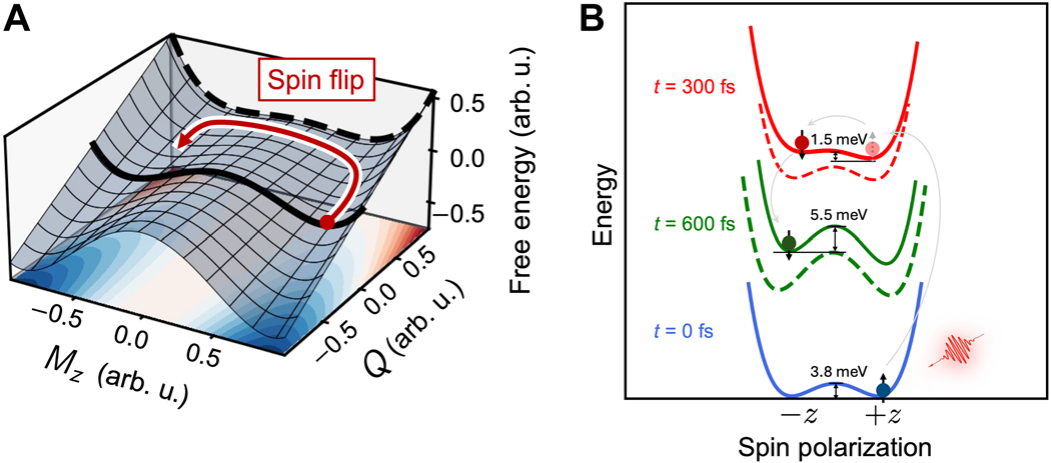
The PES illustration of light-induced spin-flip. (**A**) Energy landscape of the total effective free energy with first-order coupling. The bold black curves represent PES under equilibrium (solid) and with excited A_1g_ phonon mode (dashed), with the red arrow showing the spin-flip process, enabled by lowering the energy barrier. (**B**) Calculated PES with transient lattice structure and real-time electronic excitation, with the order parameter represented by the spin polarization rotating from +*z* to –*z* direction. The blue, red, and green solid curves correspond to the PES calculated at the chosen moment of *t* = 0 (ground state), 300, and 600 fs with TDDFT-computed electron occupation number. The red and green dashed curves represent the PES calculated with transient lattice distortions but without electronic excitation. When the pump pulse is applied, the system is stimulated to the higher PES depicted in red, where the energy barrier for the spin-flip is reduced (compared to that of the dashed line) by the nonequilibrium electron-lattice coupling. During the after-pulse dynamics, the spin-down states remain in the local minimum with an increased energy barrier (green curves).

The PES is shown in Fig. 5A. Now, we introduce interaction $F_{\text{int}}$ into the total effective free energy

$$F=F_{0}+F_{\text{int}}=\alpha M^{4}+\beta_{1}^{\prime }M_{z}^{2}+\beta_{2}^{\prime }M_{∥}^{2}+\sum_{i}\frac{1}{2}k_{i}Q_{i}^{2}+\sum_{i}\frac{1}{4}g_{i}Q_{i}^{4}$$ (3)

with ${\beta_{1}}^{\prime }=\beta_{1}+\gamma_{1}Q_{A_{1g}}$ and ${\beta_{2}}^{\prime }=\beta_{2}+\gamma_{2}Q_{A_{1g}}$ . Now, we can analyze the energy landscape of the system via the first-order perturbation. The new minima of the free energy are at $M_{z}=\pm M_{0}^{\prime }$ ($M_{∥}=0$) with

$$M_{0}^{\prime }=M_{0}\sqrt{\frac{\beta_{1}^{\prime }}{\beta_{1}}}\approx M_{0}\left(1+\frac{\gamma_{1}Q_{A_{1g}}}{2\beta_{1}}\right)$$ (4)

The energy barrier between the two minima is modified to

$$E_{b}^{\prime }=\frac{{\beta^{\prime }}_{1}^{2}-{\beta^{\prime }}_{2}^{2}}{4\alpha }\approx E_{b}-\frac{\left(\beta_{1}\gamma_{1}-\beta_{2}\gamma_{2}\right)Q_{A_{1g}}}{2\alpha }$$ (5)

As can be seen from Fig. 5A, after the excitation of the A_1g_ phonon, the energy barrier between the two minima is lowered, facilitating the spin-flip.

However, the potential energy of the double well stays degenerate in this model. In FGT, a strong laser pulse introduces spin-dependent asymmetric electronic excitation (see note S15 for details), which will break the degeneracy of energies of opposite spin polarization. Therefore, to shed more light into the nonequilibrium state, we now compute the transient excited state PES, depicted in Fig. 5B. We take the transient lattice structure and electron distribution from our TDDFT simulations at *t* = 300 fs (right after the spin-flip) and 600 fs (after stabilization of the magnetic configuration), to illustrate the variation of the energy barrier for the spin-flip. Initially, the system is positioned at one of the two local minima on the ground-state PES, with the barrier for spin-flip approximately the magnetic anisotropy energy (~3.8 meV/f.u.). As the laser is applied, the system is excited to a higher-energy PES, resulting in a 60% reduction of the potential barrier to ~1.5 meV/f.u., while pure thermal excitation will increase the barrier (see note S11 for details), consistent with the effective model. At *t* = 600 fs, energy is relaxed in electronic subsystems and transferred to the lattice accompanying nonlinear phonon interactions, leading to an increased energy barrier (~5.5 meV/f.u.) that stabilizes the spin order. In theory, one would expect that the coherent phonon generation will also modify the exchange interaction and the magnetic anisotropy. However, such manifestation coincides with the change of the Berry curvature, both correlated with the strong SOC. We highlight that this PES representation is projection of a high-dimensional multiple-variable surface, simultaneously accounting for the electron excitation (by changing the electron distribution), coherent phonon vibrations (by taking the transient lattice structure), as well as the spin order (by varying the magnetic moment). The physical picture aligns well with our nonadiabatic TDDFT simulations, pinpointing the role of displacively excited coherent phonons and the nonequilibrium electron occupation.

## DISCUSSION

As an outlook, we discuss the possibility of achieving successive spin-flips as a demonstration of spintronic device concepts such as magnetic tunneling junctions. By applying another laser pulse at ~690 fs, we observe a trend for a second-flip, although the magnitude of *M_z_* is largely reduced. This is due to unrealistic thermalization effects since the relaxation channels are limited in our current simulations. Signatures of the second-flip is significantly enhanced when lowering the fluence of both pulses and extending the time delay between them (note S9), yet we anticipate that simulations with a bigger cell size are favorable to observe successive spin-flips. It is also noteworthy from a recent work (*49*) that it is possible to switch the excitation directions of coherent phonons by varying the wavelength of the pump laser. However, this is highly dependent on the subtlety of the electronic state–resolved electron-phonon interactions that needs to be further explored.

For the magnetization dynamics we proposed, magneto-optics such as Kerr and Faraday effects are the state-of-art probe. Although pump probe–based methods can reach picosecond resolution when using visible or infrared light (*50*, *51*) using extreme ultraviolet light pulses, previous works have measured femtosecond, element-resolved ultrafast magnetization dynamics (*52*, *53*). We propose that with delicate engineering of the pulse series, i.e., duration, fluence, and temporal separation, a nonvolatile and deterministic femtosecond spin-flip can be realized and practically detected with the progress of characterization techniques.

Last, it is well established that the equilibrium Berry curvature can be probed by the macroscopic anomalous Hall effect (*54*, *55*) and dichroic angle-resolved photoelectron spectroscopy (*56*). Although characterizing the transport signal with a femtosecond resolution is challenging, the light-induced Anomalous Hall effect in graphene (*57*) makes it an intriguing direction to bridge the nonequilibrium band topology with transient macroscopic properties.

## MATERIALS AND METHODS

### Theory

The dynamics of the excited states have been effectively simulated using the time-dependent ab initio package (*33*, *34*, *58*). This computational approach was implemented within the QUANTUM ESPRESSO package (*34*, *59*–*61*). Following the principles of rt-TDDFT (*32*, *33*, *62*), the temporal evolution of the two-component Kohn-Sham spinors denoted as $\psi_{\gamma ,k}\left(r,t\right)$ is governed by the time-dependent Kohn-Sham equation (*58*, *63*, *64*)

$$i\hbar \frac{\partial }{\partial t}\psi_{\gamma ,k}\left(r,t\right)=H_{\text{KS}}\psi_{\gamma ,k}\left(r,t\right)$$ (6)

The Hamiltonian, denoted as ${\hat{H}}_{\text{KS}}$, can be expressed as follows

$$\begin{array}{cc} {\hat{H}}_{\text{KS}}= & \frac{1}{2m}{\left(-i\hbar \nabla +\frac{e}{c}A\left(t\right)\right)}^{2}+\sum_{\alpha }{\hat{v}}_{\text{pp}}\left(r-R_{\alpha }\left(t\right)\right)\\ & +{\hat{v}}_{H_{\text{XC}}}\left[\rho \left(r,t\right)\right]+\mu_{B}\hat{\sigma }\cdot \frac{\partial E_{\text{XC}}}{\partial m}+{\hat{v}}_{\text{soc}} \end{array}$$ (7)

where the kinetic energy of the electron under the time-dependent vector potential A(t) is denoted as $\frac{1}{2m}{\left(-i\hbar \nabla +\frac{e}{c}A\left(t\right)\right)}^{2}$. The sum of the local atomic potential $\sum_{\alpha }{\hat{v}}_{\text{pp}}\left(r-R_{\alpha }\left(t\right)\right)$ and the Hartree exchange correlation potential ${\hat{v}}_{H_{\text{XC}}}\left[\rho \left(r,t\right)\right]$ are included in atomic pseudopotentials. The SOC term is represented as ${\hat{v}}_{\text{soc}}$, which can be written as

$${\hat{v}}_{\text{soc}}=\frac{\hbar }{4m^{2}c^{2}}\hat{\sigma }\cdot \left(\nabla V\times \hat{p}\right)$$ (8)

Once the time-dependent Kohn-Sham wave functions are obtained, the time-dependent magnetization dynamics can be derived as below

M(t)=∫m(r,t)dr (9)

where the time-dependent local magnetization is

$$m\left(r,t\right)=\mu_{B}\sum_{n,k}\psi_{n,k}^{†}\left(r,t\right)\cdot \hat{\sigma }\cdot \psi_{n,k}\left(r,t\right)$$ (10)

with $\mu_{B}$ representing the Bohr magneton ($\mu_{B}=\frac{e\hbar }{2m}$).

The ion dynamics, described by $R_{\alpha }\left(t\right)$, are treated classically, which follows the Hellmann-Feynman theorem (*65*)

$$M_{\alpha }\frac{d^{2}}{dt^{2}}R_{\alpha }\left(t\right)=F_{\alpha }\left(t\right)$$ (11)

Here, $M_{\alpha }$ represents the mass of the α-th ion, and $R_{\alpha }$ represents its position. The instantaneous mean-field Ehrenfest forces exerted on each ion are given by

$$F_{\alpha }=-\sum_{\gamma }f_{\gamma }\langle \psi_{\gamma }|\nabla {\hat{H}}_{\text{KS}}|\psi_{\gamma }\rangle$$ (12)

where $f_{\gamma }$ is the occupation number of time-dependent Kohn-Sham wave functions $\psi_{\gamma }$.

The time evolution of dominant phonon modes can be analyzed by decomposing the atomic displacements into phonon eigenmodes $e_{\nu q}$ with wave vector q and mode ν. The atomic displacement $u_{\alpha }\left(t\right)$ is expressed as

$$u_{\alpha }\left(t\right)=\sum_{\nu q}\frac{Q_{\nu q}\left(t\right)}{\sqrt{M_{\alpha }}}e_{\nu q}^{\alpha }e^{-i\left(\omega_{\nu q}t-q\cdot R_{\alpha }-\phi_{\nu q}\right)}$$ (13)

where $Q_{\nu q}\left(t\right)$ is the amplitude of vibration, $e_{\nu q}^{\alpha }$ is the eigenvector of the phonon mode, and $\phi_{\nu q}$ is the phase factor. Given the orthogonality of phonon eigenvectors, $Q_{\nu q}\left(t\right)$ can be extracted by taking the inner product between the atomic displacements u(t) and the phonon eigenvectors $e_{\nu q}^{\alpha }$.

### Computational details

The electronic and magnetic properties of monolayer FGT have been computed using DFT within the QUANTUM ESPRESSO package (*59*, *60*). In addition, phonon frequencies and eigenvectors were determined using density functional perturbation theory (*66*). In our calculations, the generalized Perdew-Zunger local density approximation (*67*) was used for the electronic exchange-correlation contribution to the total energy (*68*). Valence electron wave functions were represented using plane-wave basis sets with an energy cutoff of 120 Ry. The core electrons and nuclei were described using full-relativistic, norm-conserving pseudopotentials (*69*, *70*) from the PseudoDojo pseudopotentials library (*71*). The Brillouin zone was sampled with a Γ-centered 11 × 11 × 1 Monkhorst-Pack *k*-point grid (*72*). The SOC effect was included in all the calculations. For accuracy, the atomic structure and positions were fully relaxed with convergence thresholds of 10^−5^ atomic units (au) for ionic forces and 10^−6^ Ry for total energy. The calculated lattice constant is 3.991 Å, and the calculated magnetic moment is ~5.02 μ_B_/f.u. in the primitive cell with the magnetization along the *z* axis. These values are in excellent agreement with previous experimental and theoretical results (*40*, *41*, *73*).

For the laser excitation, we used a Gaussian-enveloped laser pulse characterized by the following waveform

$$E\left(t\right)=E_{0}\text{cos}\left(2\pi \omega t\right)\text{exp}\left[-{\left(t-t_{0}\right)}^{2}/2\sigma^{2}\right]$$ (14)

where σ = 27.6 fs (FWHW of the laser pulse is 55.2 fs), and ℏω = 1.55 eV is the photon energy. At the moment $t_{0}=116$ fs, the laser field reaches its maximal intensity, $E_{0}=0.15$ V/Å. In our dynamical computations, we used a reduced 5 × 5 × 1 *k*-mesh, and the time step was 0.145 fs for nuclei and 0.145 as for electrons.

Tight-binding model matrix elements were calculated using Wannier90 interface (*74*), and the Berry curvature was obtained using WannierTools (*75*). Both Fe, Ge, and Te *d*-orbitals, as well as Te and Ge *p*-orbitals, were included to obtain the maximized localized Wannier functions. The calculated ground state band structure with SOC is consistent with the DFT-calculated results, shown in fig. S8. The dynamical band structure and Berry curvature in Fig. 3 and fig. S9 were computed with the transient crystal structures. Specifically, the Chern number at each moment in time is obtained as

$$C\left(t\right)=\frac{1}{2\pi }\sum_{k}\Omega \left(k,t\right)\Delta k_{x}\Delta k_{y}$$ (15)

where Ω(k,t) is the Berry curvature at each point k in the Brillouin zone and at time *t*, and $\Delta k_{x}$,$\Delta k_{y}$ denotes the momentum grid spacing. The Berry curvature is calculated using the standard formula and obtained from package WannierTools. We note that the quantity *C*(*t*) computed here is an instantaneous Chern number defined at each time snapshot and, therefore, should not be interpreted as a topological invariant of the full time evolution. Rather, following the electronic excitation, the lattice is distorted, and we take the transient lattice structure and compute the quasistatic topological invariant. Therefore, *C*(*t*) reflects the local topological character of the instantaneous band structure at time *t*, which varies dynamically. In practice, the Brillouin zone is discretized using an 11 × 11 fine mesh to ensure numerical convergence.

## Supplementary Materials

### This PDF file includes:

Notes S1 to 15 Figs. S1 to S16 Tables S1 to S4 References
