# Supplementary material for: Photoinduced femtosecond spin-flip assisted by a single-mode linear phonon
Source: Sci Adv. 2026 Feb 4;12(6):eadv9616. doi: 10.1126/sciadv.adv9616 (PMC12871463; doi:10.1126/sciadv.adv9616)
Supplement: Supplementary file 1 — Notes S1 to 15 Figs. S1 to S16 Tables S1 to S4 References [file sciadv.adv9616_sm.pdf]

Science Advances
AAAS

Supplementary Materials for

Photoinduced femtosecond spin-flip assisted by a single-mode linear phonon

Na Wu *et al.*

Corresponding author: Yaxian Wang, yaxianw@iphy.ac.cn; Sheng Meng, smeng@iphy.ac.cn

Sci. Adv. **12**, eadv9616 (2026)
DOI: 10.1126/sciadv.adv9616

This PDF file includes:

Notes S1 to 15
Figs. S1 to S16
Tables S1 to S4
References

Extended data

Supplementary Note 1: The spin-flip under intense laser

In this section, we provide details, *i.e.* the temporal evolution of the *x*-, *y*-, and *z*-components of the magnetic moment during the instantaneous spin-flip under an intense laser pulse with the fluence of 7.72 mJ/cm^2 (as detailed in the main text).

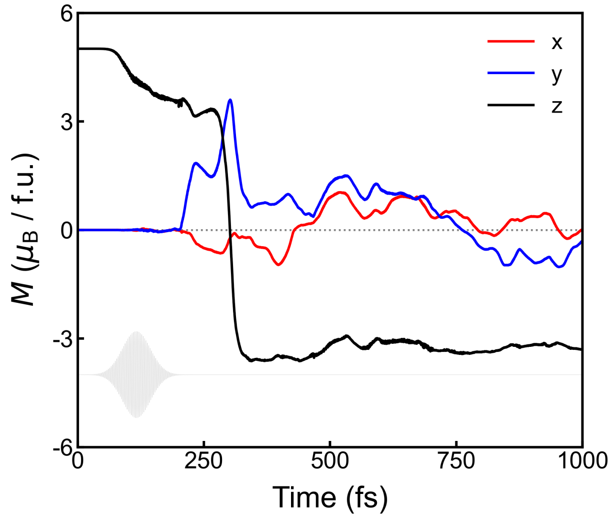

Figure S1: **The spin-flip under intense laser.** The temporal dynamics of *x*-, *y*- and *z*-components of the magnetic moment in FGT under the intense laser (fluence of 7.72 mJ/cm^2 mentioned in the main text).

Supplementary Note 2: The demagnetization under weak laser

In this section, we investigate the time-dependent magnetization dynamics under exposure to the weak laser (fluence of 1.09 mJ/cm^2), which features the demagnetization process. Figure S2 illustrates the temporal evolution of the *x*-, *y*-, and *z*-components of the magnetic moment during this demagnetization.

Supplementary Note 3: The ferromagnetic spin-melting under strong laser

In this section, we show the temporal evolution of the *x*-, *y*-, and *z*-components of magnetic moment in the ferromagnetic spin-melting regime, shown in Fig. S3.

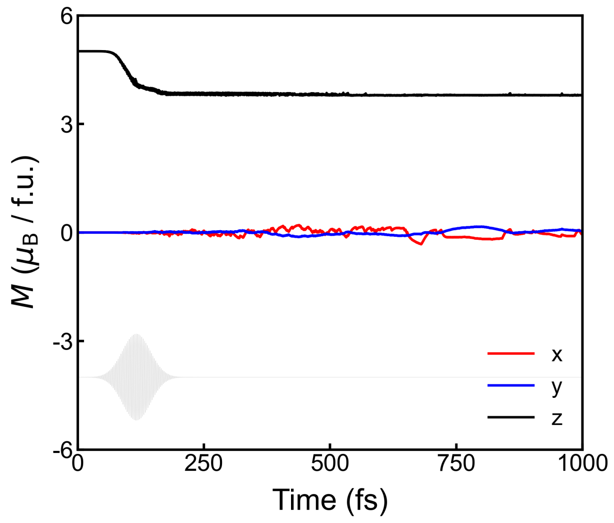

Figure S2: **The demagnetization under weak laser.** The temporal dynamics of x-, y- and z-components of the magnetic moment under a weak laser (fluence of 1.09 mJ/cm^2), as a direct comparison with the dynamics observed under the intense laser in Fig. S1.

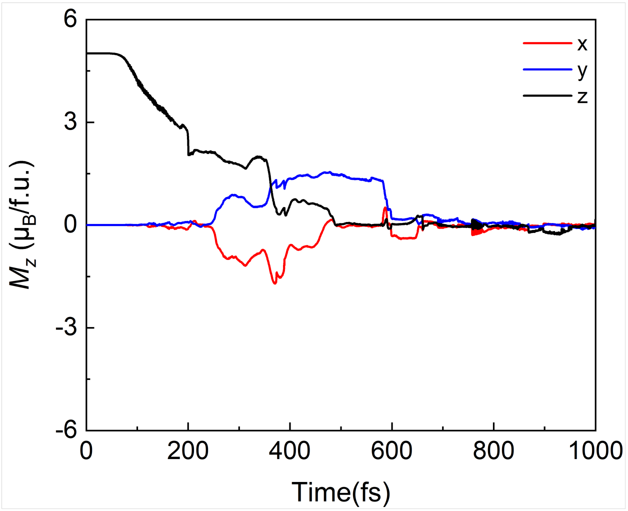

Figure S3: **The ferromagnetic spin-melting under strong laser.** The temporal dynamics of x-, y- and z-components of the magnetic moment in FGT under strong laser (fluence of 12.06 mJ/cm^2). The z-direction magnetic moment approaches zero after the critical time.

Supplementary Note 4: The role of A_{1g} coherent phonon

To further investigate the role of coherent phonons, we simulate different magnetization dynamics technically by either fixing or allowing atomic motions along the phonon eigenvectors, as illustrated in Fig. S4. Our results reveal that the femtosecond spin-flip is primarily driven by photoinduced out-

of-plane A_{1g} coherent phonons. Still, the critical time for the ultrafast spin-flip is notably affected by the presence of other phonons.

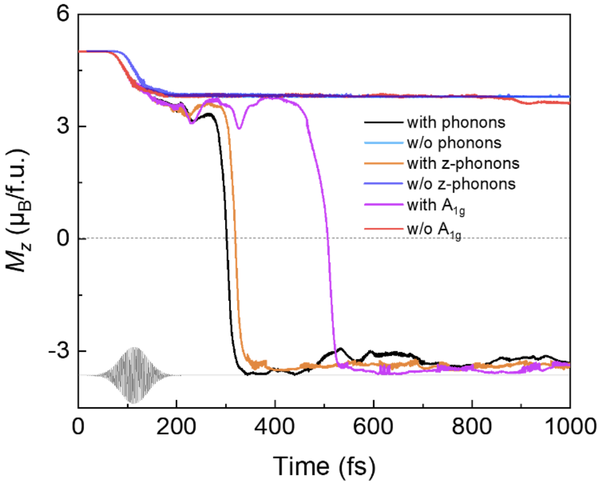

Figure S4: **The magnetization dynamics involving different lattice degrees of freedom.**

Supplementary Note 5: The role of electron excitation

To clarify the impact of electron excitation, we investigate the magnetization dynamics in the presence of coherent A_{1g} phonons but under different electronic excitations, shown in Fig. S5. We observe no spin-flip under either adiabatic Born-Oppenheimer molecular dynamics (BOMD, red curve) or nonadiabatic molecular dynamics (NAMD, blue curve), as a direct comparison with our original simulation (black curve). It is thus evident that the excited-state electron distribution has a significant effect on ultrafast spin-flip dynamics. Fig. S10 shows the transient electronic density of states and corresponding electron occupations at four different snapshots (t=0, 300, 600, and 1000 fs). The red circles represent the number of electrons occupying the energy levels, showing a strong deviation from the thermally expected Fermi-Dirac distribution.

Supplementary Note 6: Coherent-phonon renormalized bandstructure

The energy band structure of monolayer FGT with and without spin-orbit coupling (SOC) interaction is presented in Fig. S6 **a**. It can be seen that SOC induces multiple gap openings along the K-Γ high-symmetry path (we named as Q point). Fig. S6 **c** shows the distorted band structure under

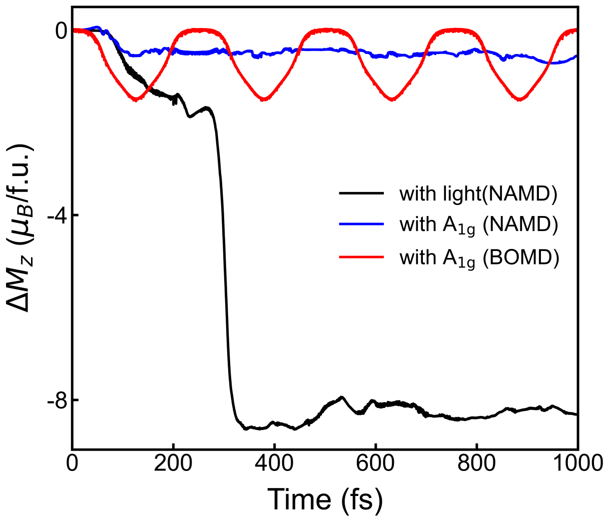

Figure S5: **The role of electron excitation.** With the presence of the A_{1g} phonon mode but no laser excitation, there is no spin-flip under both adiabatic Born-Oppenheimer (in red) and nonadiabatic molecular dynamics (in blue) simulations. The black curve is adopted from the real-time simulation in Fig. 2 in the main text.

four frozen phonon modes (A_{1g}-1, 2 and A_{2u}-1, 2), each with an amplitude of 0.1 $\sqrt{\text{amu}}\cdot$ Å. These states mostly consist of the Fe_I-d_{z^2}, d_{xz} orbitals and the Fe_{II}-$d_{x^2-y^2}$ orbital (Fig. S6 **b**), the former of which is expected to be sensitive to the out-of-plane lattice motions. It is evident that both A_{1g} modes cause rather dramatic renormalization to the states near the band crossings, much larger than one would expect for a typical metal. This indicates that for coherent phonons excited to large amplitude, the nonequilibrium electron-phonon interactions are likely to be beyond the perturbation regime.

Supplementary Note 7: The ground-state bandstructure calculated with DFT and Wannier90

The calculated ground-state band structure with Wannier90 (see *Materials and Methods* in the main text) is shown in red in Fig. S7. The result is consistent with the DFT results shown in black, verifying the reliability of our maximized localized Wannier functions (MLWFs).

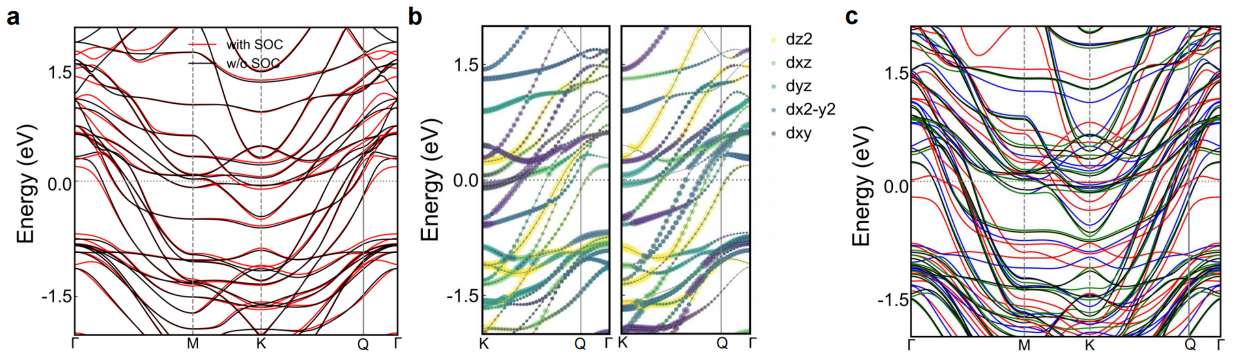

Figure S6: **Coherent-phonon renormalized bandstructure. a** The energy band structure of monolayer FGT with (in red) and without SOC (in black). There are multiple band openings along the high symmetry path. **b** Orbital projected bandstructures along the high symmetry Γ-K path. The left (right) pannel represents the PDOS of Fe_I (Fe_{II}) atom. **c** The energy band structure upon lattice distortions along four optical phonon modes, all with the same amplitude of 0.1 $\sqrt{\text{amu}}\cdot$ Å(red: A_{1g}-1, blue: A_{1g}-2, green: A_{2u}-1, yellow: A_{2u}-2), respectively. The black curve represents the initial band structure of pristine monolayer FGT.

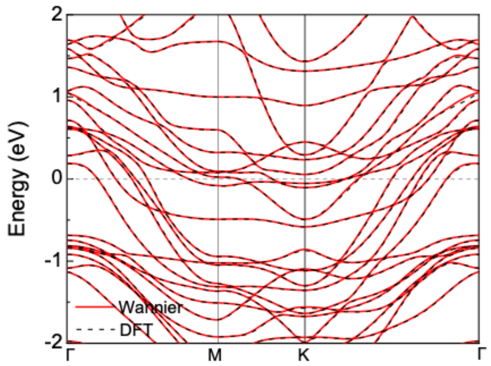

Figure S7: **The ground-state band structure of monolayer FGT.** Bandstructure calculated with DFT (black) and extrapolated from Wannier functions (red), showing great consistency.

Supplementary Note 8: The transient Berry curvature under weak excitation

In this section, we make a comparison of the transient Berry curvature under a weak excitation with laser fluence 1.09 mJ/cm^2, where no emergence of spin-flip but instead of demagnetization is observed (Fig. S2). The corresponding transient Berry curvature at the same chosen snapshots is demonstrated in Fig. S8. The Berry curvature distribution in the Brillouin zone has negligible

changes.

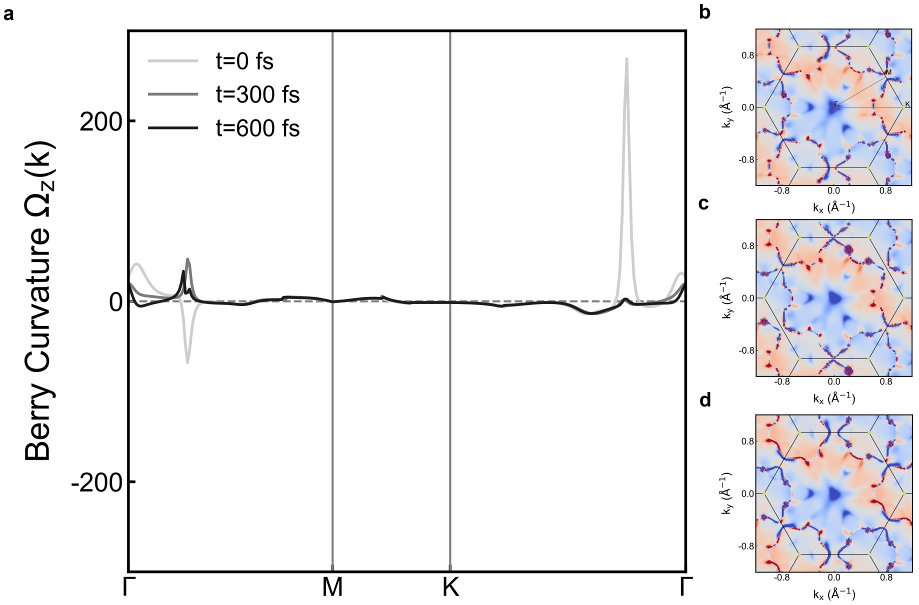

Figure S8: **The transient Berry curvature under weak excitation. a** The z-direction Berry curvature $\Omega_z(k)$ along the high-symmetry path Γ-M-K-Γ in the 2D Brillouin zone. Evolution of the Berry curvature distribution in the 2D Brillouin zone at the three chosen moment of **b** $t = 0$ fs, **c** $t = 300$ fs, and **d** $t = 600$ fs, showing negligible changes.

Supplementary Note 9: The two-pulse driven spin dynamics

In this section, we investigate the possibility of manipulating spin-flip using two successive identical pulses at approximately 116 fs and 616 fs, as shown in Fig. S9. It can be seen from Fig. S9**a** that the deterministic signature for the second spin-flip is absent, as M_z is approaching zero, mostly due to the fact that there is not energy damping to the environment. To further explore the possibility, we have tried to lower the pump fluence of both pulses, and explored the effect of time delay between the two pulses. As can be seen from Fig. S9**b**, extending the time delay between the two pulses (from 500 fs to 1000 fs) but leaving the second pulse with the same fluence results in similar melting effect, so as in the case of reducing the fluence of second pulse to 5.79 mJ/cm^2 (Fig. S9**c**). However, if we at the same time lower the fluence of the first pulse to 6.27 mJ/cm^2, we did observe a signature for a second spin-flip, shown in Fig. S9**d**. Still, we expect that performing simulations with a more realistic cell size to include more scattering phase space is favorable to observe the successive spin

flip, but the TDDFT calculation is extremely computationally expensive and we have to leave this for future work.

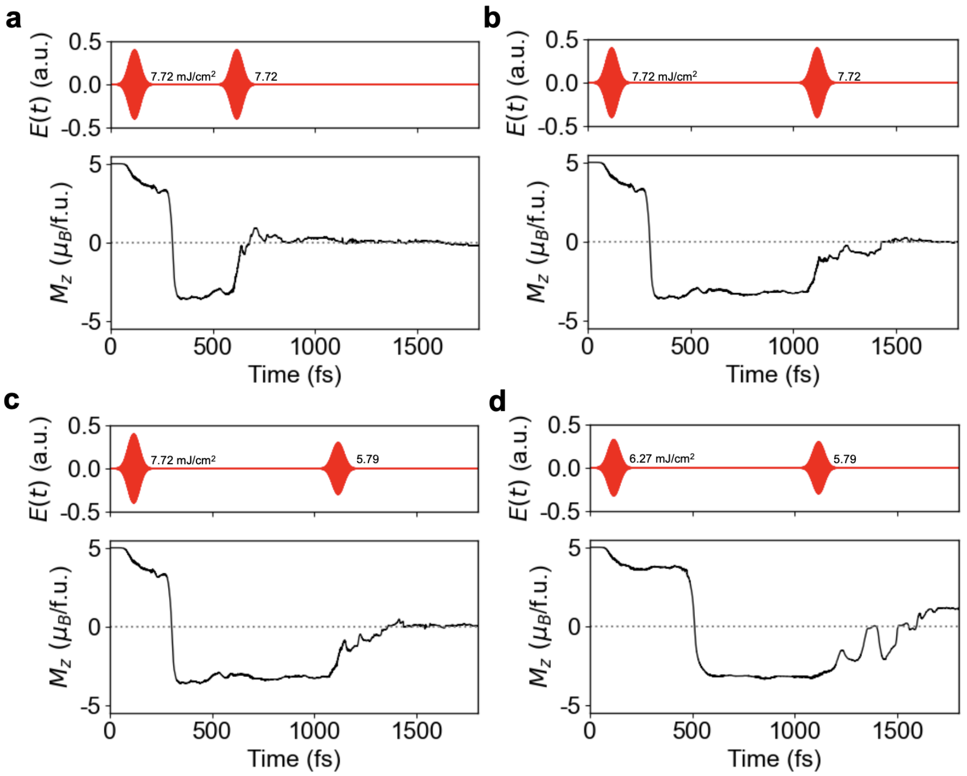

Figure S9: **The two-pulse driven spin dynamics.** Time evolution of the out-of-plane magnetization M_z under two-pulse excitation, with varying time delay and pump fluence of both pulses. When lowering the first pump fluence to 6.27 mJ/cm^2 and extending the time delay between the two pulses, signature of a second spin flip is observed in **d**.

Supplementary Note 10: Theoretical modeling of free energy and the process of spin flip

In this section, we use the phenomenological Landau-Ginzburg free-energy model to explain the spin-flip process and the coherent phonon generation in FGT via magneto-phononic coupling. The order parameters of FGT are the magnetization $\boldsymbol{M}$ and the amplitude of i-th phonon modes Q_i, as described in main text. The non-interacting anisotropic scalar free energy of FGT is expressed as:

$$F_0 = \alpha M^4 + \beta_1 M_z^2 + \beta_2 M_\parallel^2 + \sum_i \frac{1}{2} k_i Q_i^2 + \sum_i \frac{1}{4} g_i Q_i^4, \qquad \text{(S1)}$$

where $M = |\boldsymbol{M}|$, $\alpha, \beta_{1,2}$ and k_i, g_i are material parameters. M_z and $M_{||}$ denote the magnetization along and perpendicular to the easy-axis, i.e. z-axis, respectively. We assume $\beta_1 \neq \beta_2$ to describe the magnetic anisotropy. Here we choose the coordinate system with the z-axis is perpendicular to the plane of FGT, and set $|\beta_1| > |\beta_2|$ to ensure that the existence of local minimum at $M_z = \pm M_0$ and $M_{||} = 0$, where $M_0 = \sqrt{\frac{-\beta_1}{2\alpha}}$. And the energy barrier between the two minima is given by:

$$E_b = \frac{\beta_1^2 - \beta_2^2}{4\alpha}. \tag{S2}$$

To elucidate the role of phonon during spin-flip process, we incorporate the interaction terms between the magnetization and lattice vibrations derived from irreducible representations (IRREPs) of the phonon modes and the magnetization (*76*). For monolayer FGT, all optical phonon modes at Γ point decompose as:

$$\Gamma_{\text{opt}} = 2A_1' \oplus 3A_2'' \oplus 3E' \oplus 2E'' \quad \text{(15 branches in totol)} \tag{S3}$$

The correspondence between bulk and monolayer FGT symmetry representations can be deducted from the correlation of group-subgroup pairs from D_{6h} to D_{3h}, as detailed in Tab. S1. And the symmetry labels of monolayer FGT is adopted in this section.

Table S1: **Irreducible representations of the phonon modes.** The correlation relation for group-subgroup pairs from D_{6h} (bulk) to D_{3h} (monolayer) are also listed.

D_{3h}	D_{6h}
A_1'	A_{1g}, B_{2u}
A_2'	A_{2g}, B_{1u}
A_1''	A_{1u}, B_{2g}
A_2''	A_{2u}, B_{1g}
E'	E_{1u}, E_{2g}
E''	B_{1g}, E_{2u}

Without loss of clarity, we adopt the notation $M_{||}$ to label the 2D subspace of the magnetization that is parallel to the plane of FGT, i.e., (M_x, M_y). And the notation M_z is also adopted to label the subspace constituted by z-component of the magnetization. The transformation properties of axial

vector yield the representations (REPs) χ of these two magnetization components are $\chi(M_{||}) = E''$ and $\chi(M_z) = A_2'$ respectively.

A systematic symmetry analysis was performed to identify all allowed magneto-phononic coupling terms up to second order. The construction of interaction terms relies on the direct product of phonon and magnetization representations, where only products containing the identity representation A_1' are symmetry-allowed.

At linear order in phonon amplitude and magnetization ($Q \cdot M$), only one coupling term emerges:

$$\chi(Q_{E''}) \otimes \chi(M_{||}) = A_1' \oplus A_2' \oplus E'. \tag{S4}$$

We note that the E'' mode is doubly degenerate, the full degree of freedom can be expressed as $Q_{E'',x}, Q_{E'',y}$ in the Cartesian coordinate system or as $Q_{E'',R/L} = Q_{E'',x} \pm iQ_{E'',y}$ in circular basis with the complex notation.

For interaction terms with order $Q^2 \cdot M$, the direct products of the REPs which contain the identity representation A_1' are:

$$\begin{aligned}
\chi(Q_{A_1'}) \otimes \chi(Q_{E''}) \otimes \chi(M_{||}) &= A_1' \oplus A_2' \oplus E', \\
\chi(Q_{A_2''}) \otimes \chi(Q_{E'}) \otimes \chi(M_{||}) &= A_1' \oplus A_2' \oplus E', \\
\chi(Q_{E'}) \otimes \chi(Q_{E''}) \otimes \chi(M_{||}) &= A_1' \oplus A_2' \oplus 3E', \\
[\chi(Q_{E'}) \otimes \chi(Q_{E'})]_A \otimes \chi(M_z) &= A_1', \\
[\chi(Q_{E''}) \otimes \chi(Q_{E''})]_A \otimes \chi(M_z) &= A_1'.
\end{aligned} \tag{S5}$$

Here the subscript A in $[\cdots]_A$ denotes the *antisymmetric* component of the direct product, while S and $[\cdots]_S$ will later indicate the *symmetric* component. Notably, for nonlinear interactions involving E' and E'' modes, only antisymmetric combinations are symmetry-allowed.

The allowed second-order coupling terms of type $Q \cdot M^2$ are:

$$\begin{aligned}
\chi(Q_{A_1'}) \otimes [\chi(M_{||}) \otimes \chi(M_{||})]_S &= A_1' \oplus E', \\
\chi(Q_{E'}) \otimes [\chi(M_{||}) \otimes \chi(M_{||})]_S &= A_1' \oplus A_2' \oplus 2E', \\
\chi(Q_{E''}) \otimes \chi(M_{||}) \otimes \chi(M_z) &= A_1' \oplus A_2' \oplus E', \\
\chi(A_1') \otimes [\chi(M_z) \otimes \chi(M_z)]_S &= A_1'.
\end{aligned} \tag{S6}$$

For the higher order interaction terms of type $Q^2 \cdot M^2$, the symmetry analysis reveals:

$$\begin{aligned}
\chi(Q_{A_2''}) \otimes \chi(Q_{E''}) \otimes [\chi(M_\parallel) \otimes \chi(M_\parallel)]_S &= A_1' \oplus A_2' \oplus 2E', \\
[\chi(Q_{E''}) \otimes \chi(Q_{E''})]_S \otimes [\chi(M_\parallel) \otimes \chi(M_\parallel)]_S &= 2A_1' \oplus A_2' \oplus 3E', \\
\chi(A_1') \otimes \chi(Q_{E''}) \otimes \chi(M_\parallel) \otimes \chi(M_z) &= A_1' \oplus A_2' \oplus E', \\
\chi(Q_{E'}) \otimes \chi(Q_{E''}) \otimes \chi(M_\parallel) \otimes \chi(M_z) &= A_1' \oplus A_2' \oplus 3E', \\
[\chi(Q_{E''}) \otimes \chi(Q_{E''})]_S \otimes [\chi(M_z) \otimes \chi(M_z)]_S &= A_1' \oplus E'.
\end{aligned} \tag{S7}$$

Based on this systematic investigation of various order of coupling and their IRREPs, we are able to construct all interaction termes with the complete results tabulated in Table S2. Note that the A_2' and A_1'' phonon modes phonon modes are absent in FGT as given in Eq. S3 and therefore are consequently excluded from above consideration. The notation of $Q_{...}$ and $Q'_{...}$ is adopted to distinguish the phonon modes sharing the same IRREP and symmetry but distinct vibration and frequency.

The phonons we are interested in are the photoinduced large-amplitude A_1' (A_{1g} for bulk) and A_2'' (A_{2u} for bulk) modes. Thus the lowest order interaction terms related to the dynamics of spin flip process are:

$$F_{int} = \gamma_1 Q_{A_1'} M_z^2 + \gamma_2 Q_{A_1'} M_\parallel^2, \tag{S8}$$

Here $\gamma_{1,2}$ are coupling constants.

After inserting the interaction term F_{int} into the total effective free energy $F = F_0 + F_{int}$, we can analyze the energy landscape of the system via the first-order perturbation. The energy landscape is shown in Fig.5a in the main text.

$$F = F_0 + F_{int} = \alpha M^4 + \beta_1' M_z^2 + \beta_2' M_\parallel^2 + \sum_i \frac{1}{2} k_i Q_i^2 + \sum_i \frac{1}{4} g_i Q_i^4. \tag{S9}$$

with $\beta_1' = \beta_1 + \gamma_1 Q_{A_1'}, \beta_2' = \beta_2 + \gamma_2 Q_{A_1'}$. The new minima of the free energy are at $M_z = \pm M_0', M_\parallel = 0$ with:

$$M_0' = M_0 \sqrt{\frac{\beta_1'}{\beta_1}} \approx M_0 \left(1 + \frac{\gamma_1 Q_{A_1'}}{2\beta_1}\right). \tag{S10}$$

The energy barrier between the two minima is modified to:

$$E_b' = \frac{\beta_1'^2 - \beta_2'^2}{4\alpha} \approx E_b - \frac{(\beta_1\gamma_1 - \beta_2\gamma_2) Q_{A_1'}}{2\alpha}. \tag{S11}$$

Table S2: **Symmetry-allowed interaction terms between the phonon modes and the magnetization.** The table includes coupling terms up to the second order, with out-of-plane phonon highlighted with blue color.

Order	Interaction term
$Q \cdot M$	$Q_{E'',x}M_x + Q_{E'',y}M_y$
$Q^2 \cdot M$	$Q_{A_1'}(Q_{E'',x}M_x + Q_{E'',y}M_y)$
	$Q_{A_2''}(Q_{E',x}M_x + Q_{E',y}M_y)$
	$M_x(Q_{E',x}Q_{E'',x} - Q_{E',y}Q_{E'',y}) - M_y(Q_{E',x}Q_{E'',y} + Q_{E',y}Q_{E'',x})$
	$M_z(Q_{E',x}Q'_{E',y} - Q_{E',y}Q'_{E',x})$
	$M_z(Q_{E'',x}Q'_{E'',y} - Q_{E'',y}Q'_{E'',x})$
$Q \cdot M^2$	$Q_{A_1'}M_z^2,\ Q_{A_1'}M_{\parallel}^2$
	$M_z(M_xQ_{E'',y} - M_yQ_{E'',x})$
	$(M_x^2 - M_y^2)Q_{E',x} - 2M_xM_yQ_{E',y}$
$Q^2 \cdot M^2$	$Q_{A_2''}((M_x^2 - M_y^2)Q_{E'',x} - 2M_xM_yQ_{E'',y})$
	$M_zQ_{A_1'}(M_xQ_{E'',y} - M_yQ_{E'',x})$
	$M_z(M_x(Q_{E',x}Q_{E'',y} + Q_{E',y}Q_{E'',x}) + M_y(Q_{E',x}Q_{E'',x} - Q_{E',y}Q_{E'',y}))$
	$M_z^2(Q_{E'',x}Q'_{E'',x} + Q_{E'',y}Q'_{E'',y})$
	$M_L^2Q_{E'',R}Q'_{E'',R} + \text{h.c.},\ M_LM_RQ_{E'',L}Q'_{E'',R} + \text{h.c.}$

The phonon assisted spin flip process can be understood as follows. After the laser excitation, the total energy of FGT is higher than ground state, happens with the excitation of the A_1' (A_{1g} for bulk) mode and the demagnetization of the system as given in Fig. 5 in the main text. After the excitation of the A_1' (A_{1g} for bulk) mode, the energy barrier between the two minima is lowered. This allows the magnetization to flip more easily. This is confirmed by the PES calculation from first principles, shown in Table S3.

Table S3: **Calculated energies on the potential energy surface (PES).** The energy of the double well and the barrier peak calculated from first-principles is listed.

Condition	$-M_0$ (meV)	$+M_0$ (meV)	Barrier Peak (meV)
0 fs (ground state)	0	0	3.79
300 fs (w/ electron excitation)	246.36	245.87	1.53
300 fs (w/o electron excitation)	246.55	245.62	2.14
600 fs (w/ electron excitation)	109.80	104.29	5.51
600 fs (w/o electron excitation)	109.06	102.92	6.14

Finally, we note that the out of plane mode A_2'' (A_{2u} for bulk) is excited after the spin flip process. This excitation arises from the coupling terms listed in Tab. S2 containing A_2'' mode, which are $Q_{A_2''}(Q_{E',x}M_x + Q_{E',y}M_y)$ and $Q_{A_2''}((M_x^2 - M_y^2)Q_{E'',x} - 2M_xM_yQ_{E'',y})$.

Supplementary Note 11: Discussion on thermal and nonthermal effect

In this session, we discuss how our study achieves a totally different scenario than the thermal excitation. Then, we try our best to estimate the thermal effect by computing the potential energy surface with different smearing factors.

In fact, the spin dynamics may be strongly affected by thermal effect, and a lot of the early experimental works attribute the ultrafast demagnetization and spin reversal to the heating effect from the laser pulse. However, in our TDDFT simulation, we are trying to model another extreme condition, that is, we eliminate the thermal excitation by setting zero temperature, and use a unit cell to confine the phonon scattering phase space. In this sense, the carrier and phonon distribution

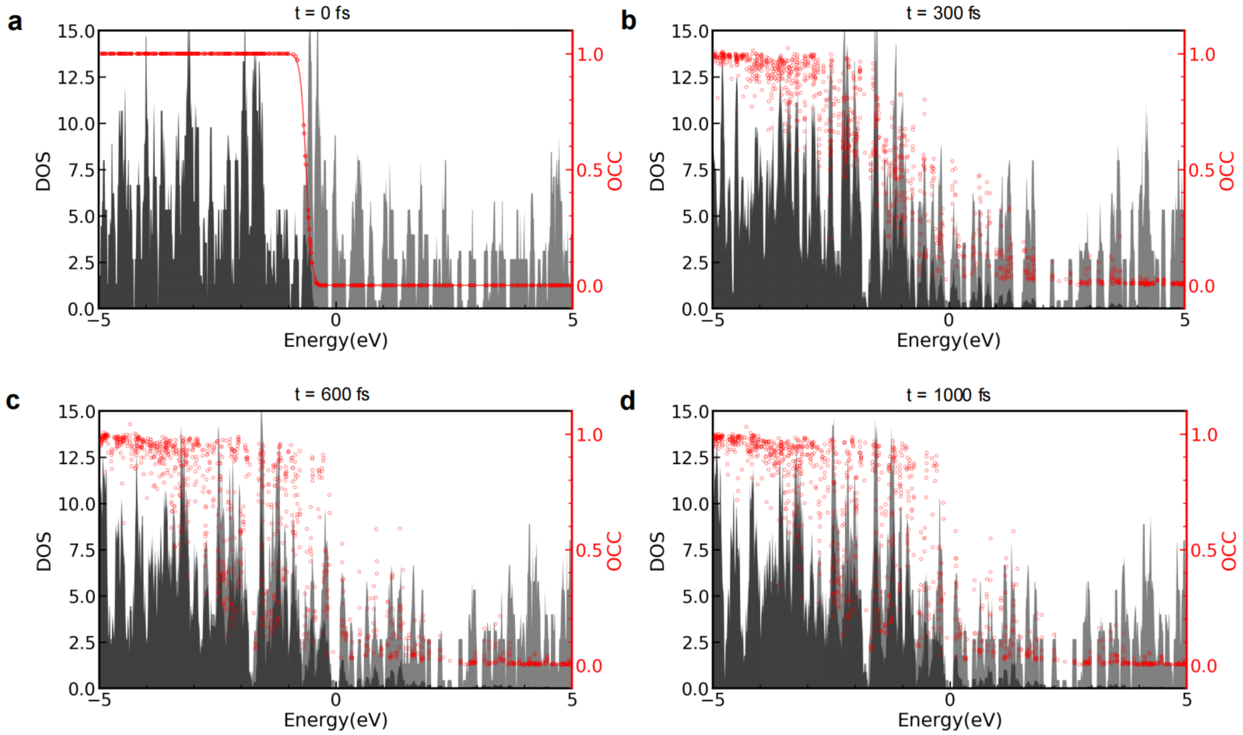

Figure S10: **Transient density of states (DOS) and corresponding electronic occupations.** Instantaneous electronic density of states and the associated electronic occupations at four distinct snapshots (t = 0, 300, 600, and 1000 fs). Red circles denote the electron occupations of each energy level. **a** At the initial moment, the electron distribution follows the Fermi-Dirac distribution. At the selected moment of **b** t = 300 fs, **c** t = 600 fs, and **d** t = 1000 fs, the electron is significantly excited, deviating far from the quasi-equilibrium Fermi-Dirac distribution.

should deviate significantly from the Fermi-Dirac and the Bose-Einstein distribution, for which one can already see in the transient electronic occupations (Fig. S10).

Regardless, we try to characterize the evolution of the electronic subsystem by extracting the time-dependent energy levels and occupation numbers of the time-evolving Kohn-Sham states and performing a self-consistent fitting using a Fermi-Dirac function. This enables us to estimate the instantaneous electronic temperature T_e:

$$f(\varepsilon) = \frac{1}{\exp\left(\frac{\varepsilon-\mu}{k_B T_e}\right) + 1}, \tag{S12}$$

where μ is the chemical potential, k_B is the Boltzmann constant, and T_e is the instantaneous electronic

temperature, shown in Fig. S11. One can see that upon photoexcitation, the effective T_e can reach a few thousand Kelvin, for which we use different electronic smearing factor to account for. Fig. S11**g** shows the modified PES and peak barrier upon increasing thermal excitation. However, the peak barrier increases instead of decreases, not favoring the spin flip process. It is hence clear that only increasing T_e but retaining the Fermi-Dirac distribution is not sufficient to induce the observed spin-flip. This also highlights the importance of taking into account the realistic excitation channel in our TDDFT simulation.

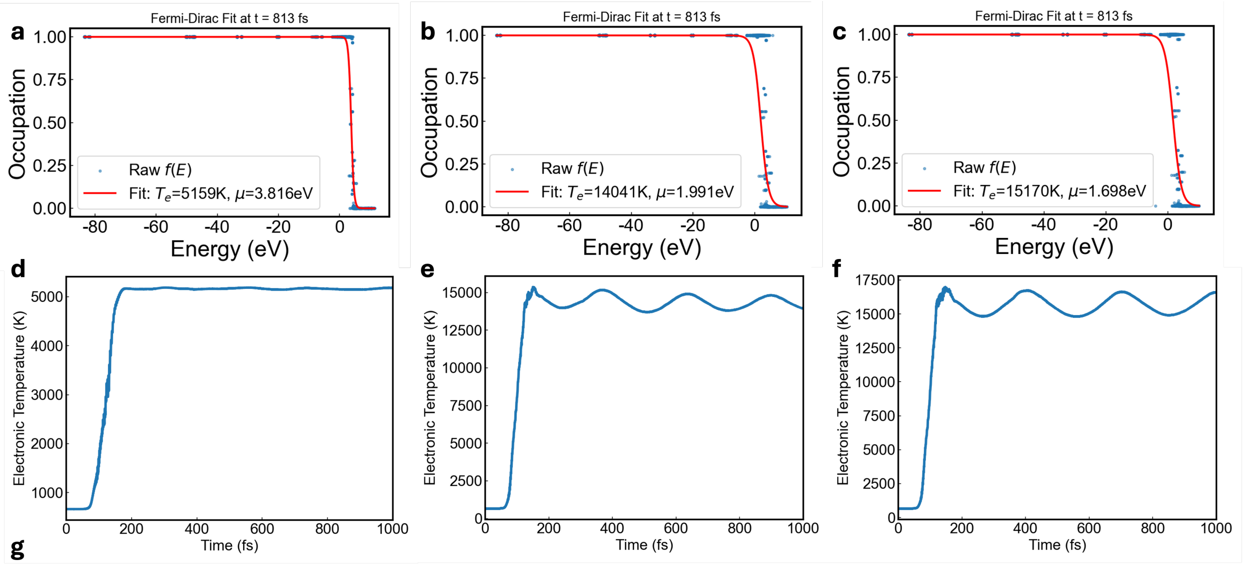

g

Smearing (Ry)	Effective Temperature (K)	$-M_0$ (meV)	M_0 (meV)	Barrier Peak (meV)	Well energy difference (meV)
degauss=1e-4	15.7	0.46	0	3.79	0.46
degauss=1e-3	157	-0.52	-0.21	4.18	0.31
degauss=1e-2	1570	-27.36	-26.91	4.33	0.45

Figure S11: **The laser induced thermalization effect. a-c** A Fermi-Dirac distribution fitting of the electronic occupation after photoexcitation under **a** demagnetization, **b** spin-flip, and **c** spin-melting regimes. **d-f** Evolution of the effective electronic temperature T_e. Higher pump fluence excites the carriers to much higher energy level, giving rise to a significant increase in T_e. **g** Calculated PES and barrier peak at different electronic smearing, which effectively mimic the thermal excitation. Unlike the *nonthermal* excitation shown in the main text, the energy barrier increases, not favoring the spin flip.

Supplementary Note 12: Time evolution of bandstructure and Berry curvature

In this note, we calculate the bandstructure at a few snapshots, shown in Fig. S12. One can see that the electronic wavefunction evolves independently at each k-wavevector. With the real-time molecular dynamics, the bandstructure is also correlated with the transient lattice structure. In Fig. S12 we show the bandstructure at each time snapshot of 0, 300, and 600 fs. It can be seen that each wavevector undergoes band closing and opening so the Berry curvature associated with a gap may have shifted, disappeared, or appeared at a different wavevector. Meanwhile, we calculate

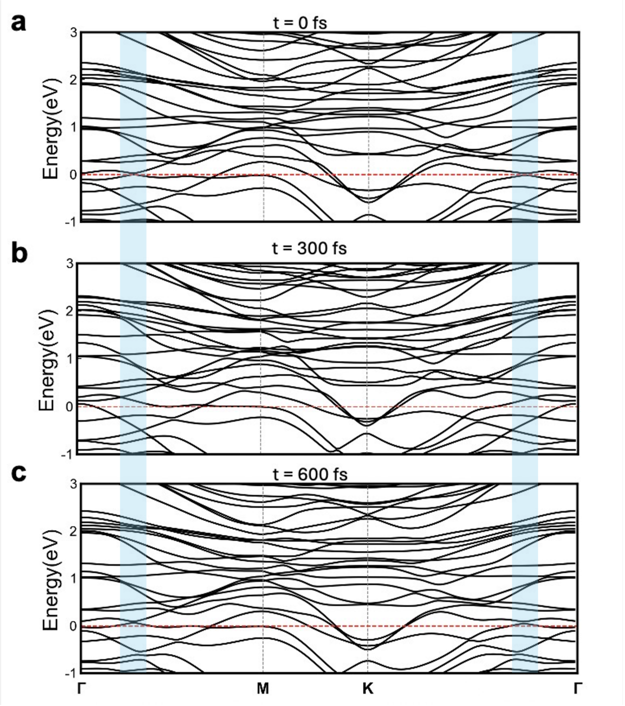

Figure S12: **The transient bandstructure.** Bandstructure for three snapshots, i.e **a** $t = 0$, **b** $t = 300$, **c** $t = 600$ fs. The wavevectors at which the Berry curvature originally peaked is show in blue shading to guide the eye.

the Berry curvature at a few snapshots, shown in Fig. S13. Similar to the Chern number evolution (Fig. 4b in the main text), we only observe a sign change, rather than an oscillatory behavior. If one can selectively excite one phonon mode, an oscillation is more likely to be observed.

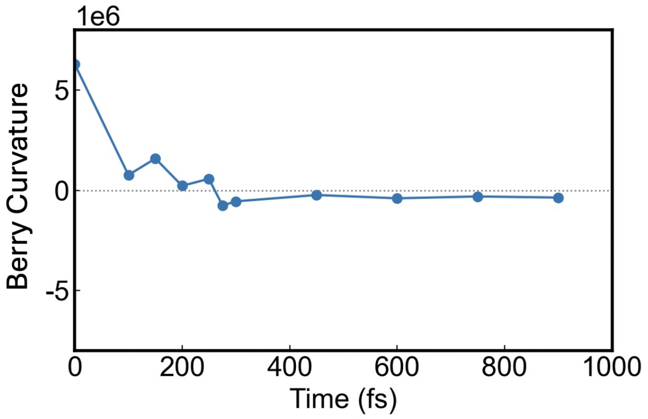

Figure S13: **The real-time evolution of calculated Berry curvature.**

Supplementary Note 13: Discussion on possible magnon excitation

To analyze possible magnon excitation, we first construct a minimal three-sublattice spin model of Fe_3GeTe_2:

$$H = -\sum_{i,j} J_{ij}\boldsymbol{S}_i\boldsymbol{S}_j - \sum_i K_i(t)(S_i^z)^2 \tag{S13}$$

where the total spin Hamiltonian of the system includes exchange interactions J_{ij} among three inequivalent magnetic sublattices (Fe_1, Fe_2, and Fe_3) and the magnetic anisotropy energy. Also the Hamiltonian in Eq. S13 can be written as the matrix form:

$$H = \begin{pmatrix} \epsilon_1 & J_{12}\sqrt{\boldsymbol{S_1S_2}} & J_{13}\sqrt{\boldsymbol{S_1S_3}} \\ J_{12}\sqrt{\boldsymbol{S_1S_2}} & \epsilon_2 & J_{23}\sqrt{\boldsymbol{S_2S_3}} \\ J_{13}\sqrt{\boldsymbol{S_1S_3}} & J_{23}\sqrt{\boldsymbol{S_2S_3}} & \epsilon_3 \end{pmatrix}. \tag{S14}$$

From the expression, we can see that the diagonal term $H_{ii} = 2K_iS_i^z + \sum_j J_{ij}\boldsymbol{S_j}$ denotes the energy cost of the magnon excitation and the off-diagonal term $H_{ij} = -J_{ij}\sqrt{\boldsymbol{S_iS_j}}$ represents the transition among different magnon modes. We employ the four-states energy mapping method, and obtain from first principles calculations the site-resolved magnetocrystalline anisotropy energies (MAE), i.e. $K_{1,0} = K_{2,0} = 0.968$ meV and $K_{3,0} = 0.544$ meV. By diagonalizing the Hamiltonian, we obtained the eigenfrequencies and eigenvectors of the three magnon modes, as summarized in Table S4. The eigenvalues are in reasonable agreement with a previous study [PHYSICAL REVIEW B 102, 014450 (2020)]. The three magnon modes exhibit distinct spatial symmetry

Table S4: **Magnon eigenmodes in in monolayer Fe_3GeTe_2.** The eigenfrequencies and eigenvectors at Γ point are listed.

Mode	Eigenvalue (meV)	Eigenvector (Fe1, Fe2, Fe3)	Feature
1	3.22	(0.62, 0.62, 0.47)	acoustic
2	264.27	(0.70, −0.70, 0.00)	non-bonding
3	403.37	(−0.33, −0.33, 0.88)	optical

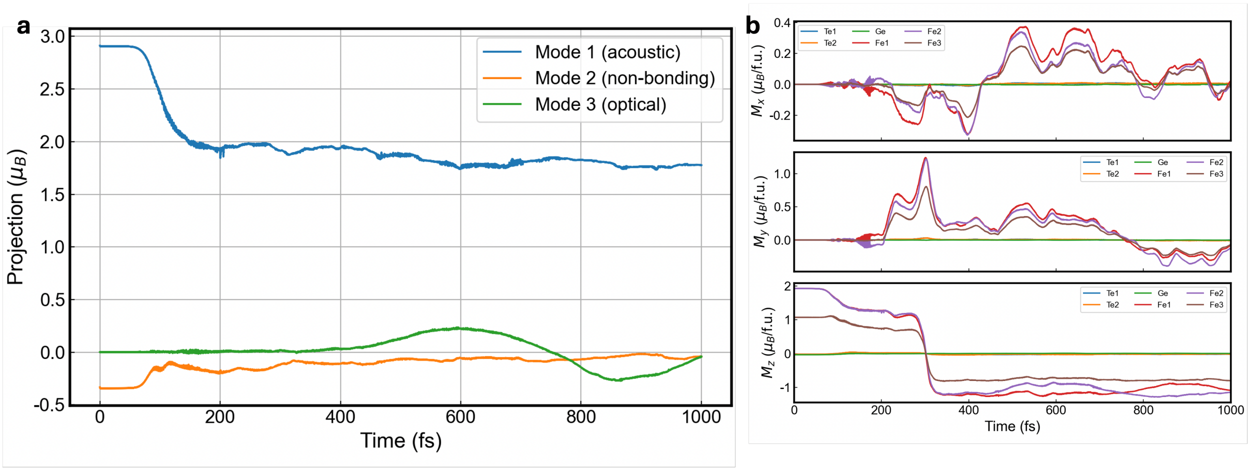

Figure S14: **The real-time magnon dynamics. a** Projected amplitude of the three magnon modes. **b** The real-time evolution of the x-, y-, and z-component of magnetization on all atoms.

features. Mode 1 corresponds to nearly in-phase oscillations of all three Fe atoms with similar amplitudes. Mode 2 shows an anti-phase motion between Fe_1 and Fe_2, while Fe_3 remains inactive. Mode 3 is characterized by a dominant out-of-phase motion of Fe_3 with respect to Fe_1 and Fe_2. These patterns reveal the cooperative spin dynamics governed by both the magnetic anisotropy and exchange interactions in the triple Fe sublattice.

Now we mimic the analysis on coherent phonon excitation, and project the real-time magnetization on the three Fe atoms onto the magnon eigenvectors. As can be seen in Fig. S14**a**, the acoustic mode (with nearly in-phase moments) dominates within the entire time span. Upon photoexcitation, there is an substantial amplitude decrease for both mode 1 and mode 2, after which they remain almost unchanged. We note that at the moment of spin-flip, say 300 fs, we do not observe any abrupt change on the magnon amplitude. However, since 400 fs, there is an excitation of the optical magnon mode, for which the timing coincides with the excitation of the A_{2u} modes. When tracking the x-,

y-, and z-component separately (Fig. S14**b**), we see Te and Ge remains nonmagnetic for the entire time, and the sign reversal for M_z happens at the same time for the three Fe atoms, accompanying an increase in the in-plane components. Overall, we do not observe a critical role of magnons in the spin-flip process, but we anticipate they are strongly correlated with the A_{2u} phonon excitation, consistent with our symmetry analysis (Supplementary Note 10). Nevertheless, since our model encodes only the phase angle of the three Fe atoms instead of the spin vector, further study is still needed to draw concrete conclusions.

Supplementary Note 14: Discussion on the A_{2u} phonon excitation

To examine the possible mechanism from nonlinear phonon coupling with the displacively excited A_{1g} phonons, we now launch the A_{1g} phonons and perform an ab initio molecular dynamics calculation. In other words, we initialize the lattice with a velocity following either A_{1g}-1 or A_{1g}-2 phonon, but avoid the carrier excitation and the photo-induced spin dynamics. As can be seen in Fig. S15, the A_{2u} modes generated have a negligible amplitude, two orders magnitude smaller than what we observed in Fig. 2 in the main text. Therefore, it is highly likely that the A_{2u} modes are launched due to the spin dynamics. For the direct spin-phonon in FGT, our previous work [Nat. Commun. 15, 2084 (2024)] shows that A_{1g} modes couple strongly to the M_z but others do not. However, our symmetry analysis indicate the inplane magnetization during the spin flip can couple to the A_{2u} modes and serve as a probable reason to drive them. Still, further study is needed to fully address the magnon-phonon coupling.

Supplementary Note 15: Discussion on the spin-dependent electronic excitation

Indeed, the fact that monolayer FGT is ferromagnetic and metallic, is essential for the symmetry breaking discussed in the main text. Since we considered spin-orbit coupling (SOC) in our calculation, the energy splitting of the spin-up and spin-down channels is not clearly visible from the band structure. To show the spin-selective excitation, we now compute the spin-resolved projected band occupations from time-dependent density functional theory (TDDFT) simulations. At each time delay (here shown for $t = 300$ fs), the real-time evolved Kohn?Sham wavefunctions $\psi_{n\mathbf{k}}(t)$ are

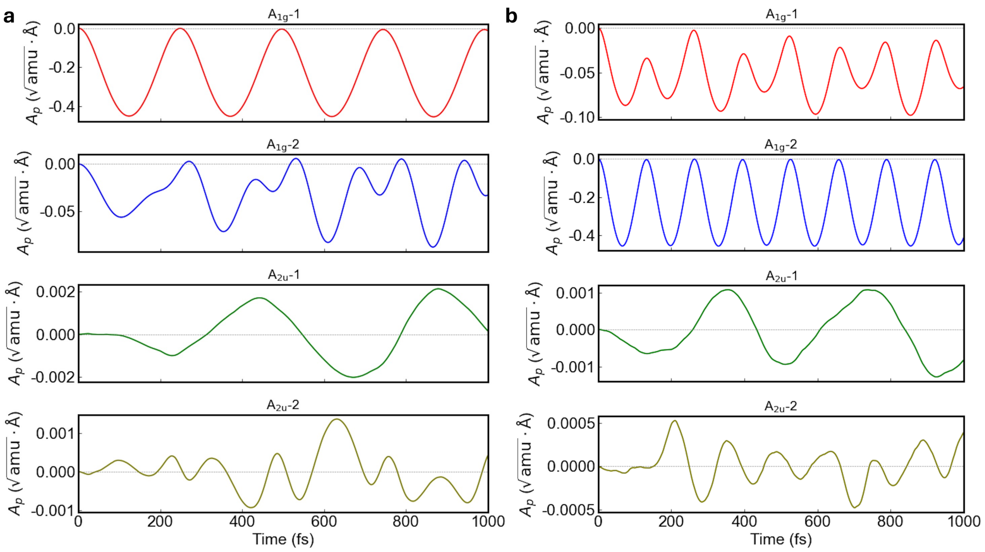

Figure S15: **Nonlinear phononics for A_{2u} modes.** Generation of A_{2u} phonons via nonlinear phonon coupling with **a** the A_{1g}-1 and **b** A_{1g}-2 mode, based on an ab initio molecular dynamics (AIMD) simulation without the photoexcited carriers.

projected onto spin-up and spin-down basis states using the projection operator

$$P_{n\mathbf{k}}^{\uparrow,\downarrow}(t) = \langle \psi_{n\mathbf{k}}(t) | \hat{P}_{\uparrow,\downarrow} | \psi_{n\mathbf{k}}(t) \rangle, \tag{S15}$$

where $\hat{P}_{\uparrow,\downarrow} = \frac{1}{2}(1 \pm \sigma_z)$. The resulting occupation weights are then plotted in momentum space, as shown in Fig. S16. The figure clearly reveals a population imbalance, which will result in nondegenerate energies on the PES.

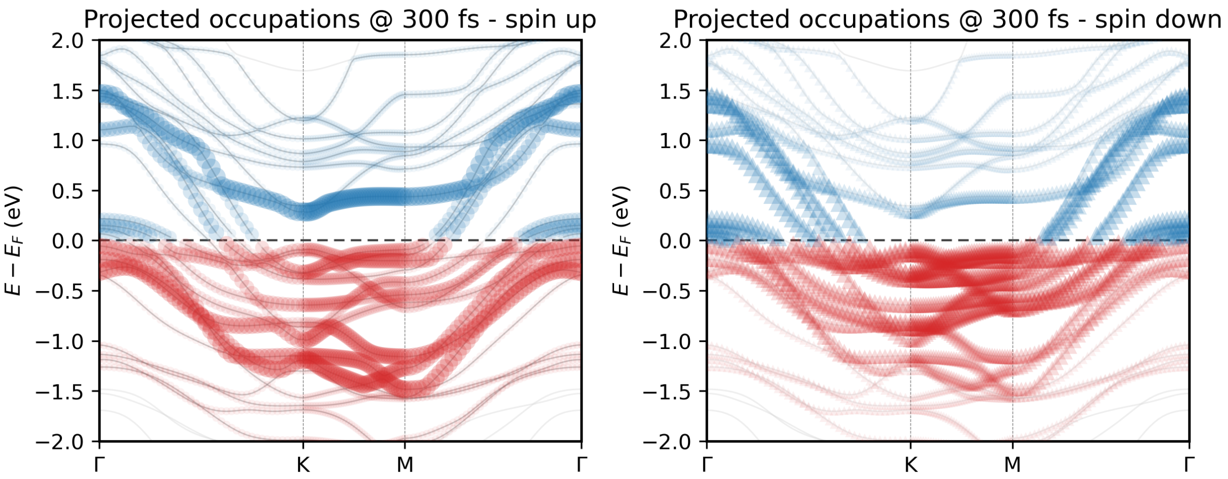

Figure S16: The spin-resolved projected electronic occupations of Fe_3GeTe_2 at $t = 300$ fs after optical excitation. The left (right) panel shows the spin-up (down) channel. The size and color intensity of the markers indicate the occupation probability projected onto each spin state, with blue (red) denoting the excited electrons (holes).

REFERENCES

1. A. S. Disa, J. Curtis, M. Fechner, A. Liu, A. von Hoegen, M. Först, T. F. Nova, P. Narang, A. Maljuk, A. V. Boris, B. Keimer, A. Cavalleri, Photo-induced high-temperature ferromagnetism in $YTiO_3$. *Nature* **617**, 73–78 (2023).

2. B. Dieny, I. L. Prejbeanu, K. Garello, P. Gambardella, P. Freitas, R. Lehndorff, W. Raberg, U. Ebels, S. O. Demokritov, J. Akerman, A. Deac, P. Pirro, C. Adelmann, A. Anane, A. V. Chumak, A. Hirohata, S. Mangin, S. O. Valenzuela, M. C. Onbaşlı, M. d'Aquino, G. Prenat, G. Finocchio, L. Lopez-Diaz, R. Chantrell, O. Chubykalo-Fesenko, P. Bortolotti, Opportunities and challenges for spintronics in the microelectronics industry. *Nat. Electron.* **3**, 446–459 (2020).

3. R. A. Leenders, D. Afanasiev, A. V. Kimel, R. V. Mikhaylovskiy, Canted spin order as a platform for ultrafast conversion of magnons. *Nature* **630**, 335–339 (2024).

4. E. Beaurepaire, J.-C. Merle, A. Daunois, J.-Y. Bigot, Ultrafast spin dynamics in ferromagnetic nickel. *Phys. Rev. Lett.* **76**, 4250–4253 (1996).

5. X. Lu, Z. Lin, H. Pi, T. Zhang, G. Li, Y. Gong, Y. Yan, X. Ruan, Y. Li, H. Zhang, L. Li, L. He, J. Wu, R. Zhang, H. Weng, C. Zeng, Y. Xu, Ultrafast magnetization enhancement via the dynamic spin-filter effect of type-II Weyl nodes in a Kagome ferromagnet. *Nat. Commun.* **15**, 2410 (2024).

6. P. Padmanabhan, F. L. Buessen, R. Tutchton, K. W. C. Kwock, S. Gilinsky, M. C. Lee, M. A. McGuire, S. R. Singamaneni, D. A. Yarotski, A. Paramekanti, J.-X. Zhu, R. P. Prasankumar, Coherent helicity-dependent spin-phonon oscillations in the ferromagnetic van der Waals crystal CrI_3. *Nat. Commun.* **13**, 4473 (2022).

7. T. Zalewski, A. Maziewski, A. V. Kimel, A. Stupakiewicz, Ultrafast all-optical toggle writing of magnetic bits without relying on heat. *Nat. Commun.* **15**, 4451 (2024).

8. N. Biniskos, F. J. dos Santos, M. dos Santos Dias, S. Raymond, K. Schmalzl, P. Steffens, J. Persson, N. Marzari, S. Blügel, S. Lounis, T. Brückel, An overview of the spin dynamics of antiferromagnetic Mn_5Si_3. *APL Mater.* **11**, 081103 (2023).

9. F. Dirnberger, J. Quan, R. Bushati, G. M. Diederich, M. Florian, J. Klein, K. Mosina, Z. Sofer, X. Xu, A. Kamra, F. J. García-Vidal, A. Alú, V. M. Menon, Magneto-optics in a van der Waals magnet tuned by self-hybridized polaritons. *Nature* **620**, 533–537 (2023).

10. P. Zhang, T.-F. Chung, Q. Li, S. Wang, Q. Wang, W. L. B. Huey, S. Yang, J. E. Goldberger, J. Yao, X. Zhang, All-optical switching of magnetization in atomically thin CrI_3. *Nat. Mater.* **21**, 1373–1378 (2022).

11. N. Wu, S. Zhang, D. Chen, Y. Wang, S. Meng, Three-stage ultrafast demagnetization dynamics in a monolayer ferromagnet. *Nat. Commun.* **15**, 2804 (2024).

12. W. Zhang, T. Ma, B. K. Hazra, H. Meyerheim, P. Rigvedi, Z. Yin, A. K. Srivastava, Z. Wang, K. Gu, S. Zhou, S. Wang, S.-H. Yang, Y. Guan, S. S. P. Parkin, Current-induced domain wall motion in a van der Waals ferromagnet Fe_3GeTe_2. *Nat. Commun.* **15**, 4851 (2024).

13. D. Afanasiev, J. R. Hortensius, B. A. Ivanov, A. Sasani, E. Bousquet, Y. M. Blanter, R. V. Mikhaylovskiy, A. V. Kimel, A. D. Caviglia, Ultrafast control of magnetic interactions via light-driven phonons. *Nat. Mater.* **20**, 607–611 (2021).

14. Y. Gao, X. Jiang, Z. Qiu, J. Zhao, Photoexcitation induced magnetic phase transition and spin dynamics in antiferromagnetic $MnPS_3$ monolayer. *npj Comput. Mater.* **9**, 107 (2023).

15. J.-Y. Bigot, M. Vomir, E. Beaurepaire, Coherent ultrafast magnetism induced by femtosecond laser pulses. *Nat. Phys.* **5**, 515–520 (2009).

16. L. Gao, S. Prokhorenko, Y. Nahas, L. Bellaiche, Dynamical multiferroicity and magnetic topological structures induced by the orbital angular momentum of light in a nonmagnetic material. *Phys. Rev. Lett.* **131**, 196801 (2023).

17. B. Koopmans, G. Malinowski, F. Dalla Longa, D. Steiauf, M. Fähnle, T. Roth, M. Cinchetti, M. Aeschlimann, Explaining the paradoxical diversity of ultrafast laser-induced demagnetization. *Nat. Mater.* **9**, 259–265 (2010).

18. G. P. Zhang, W. Hübner, Laser-induced ultrafast demagnetization in ferromagnetic metals. *Phys. Rev. Lett.* **85**, 3025–3028 (2000).

19. Z. Zheng, Q. Zheng, J. Zhao, Spin-orbit coupling induced demagnetization in Ni: Ab initio nonadiabatic molecular dynamics perspective. *Phys. Rev. B* **105**, 085142 (2022).

20. C. von Korff Schmising, S. Jana, O. Zülich, D. Sommer, S. Eisebitt, Direct versus indirect excitation of ultrafast magnetization dynamics in FeNi alloys. *Phys. Rev. Res.* **6**, 013270 (2024).

21. S. A. Ryan, P. C. Johnsen, M. F. Elhanoty, A. Grafov, N. Li, A. Delin, A. Markou, E. Lesne, C. Felser, O. Eriksson, H. C. Kapteyn, O. Grånäs, M. M. Murnane, Optically controlling the competition between spin flips and intersite spin transfer in a Heusler half-metal on sub-100-fs time scales. *Sci. Adv.* **9**, eadi1428 (2023).

22. P. A. Pantazopoulos, J. Feist, F. J. García-Vidal, A. Kamra, Unconventional magnetism mediated by spin-phonon-photon coupling. *Nat. Commun.* **15**, 4000 (2024).

23. K. Ohe, H. Shishido, M. Kato, S. Utsumi, H. Matsuura, Y. Togawa, Chirality-induced selectivity of phonon angular momenta in chiral quartz crystals. *Phys. Rev. Lett.* **132**, 056302 (2024).

24. C. S. Davies, F. G. N. Fennema, A. Tsukamoto, I. Razdolski, A. V. Kimel, A. Kirilyuk, Phononic switching of magnetization by the ultrafast Barnett effect. *Nature* **628**, 540–544 (2024).

25. A. Stupakiewicz, C. S. Davies, K. Szerenos, D. Afanasiev, K. S. Rabinovich, A. V. Boris, A. Caviglia, A. V. Kimel, A. Kirilyuk, Ultrafast phononic switching of magnetization. *Nat. Phys.* **17**, 489–492 (2021).

26. A. Hoffmann, Spin transport modified by magnetic order. *J. Magn. Magn. Mater.* **563**, 169896 (2022).

27. T. Matsuda, T. Higo, T. Koretsune, N. Kanda, Y. Hirai, H. Peng, T. Matsuo, N. Yoshikawa, R. Shimano, S. Nakatsuji, R. Matsunaga, Ultrafast dynamics of intrinsic anomalous Hall effect in the topological antiferromagnet Mn_3Sn. *Phys. Rev. Lett.* **130**, 126302 (2023).

28. A. Kimel, A. Zvezdin, S. Sharma, S. Shallcross, N. De Sousa, A. Garcia-Martín, G. Salvan, J. Hamrle, O. Stejskal, J. McCord, S. Tacchi, G. Carlotti, P. Gambardella, G. Salis, M. Münzenberg, M. Schultze, V. Temnov, I. V. Bychkov, L. N. Kotov, N. Maccaferri, D. Ignatyeva, V. Belotelov, C. Donnelly, A. H. Rodriguez, I. Matsuda, T. Ruchon, M. Fanciulli, M. Sacchi, C. R. Du, H. Wang, N. P. Armitage, M. Schubert, V. Darakchieva, B. Liu, Z. Huang, B. Ding, A. Berger, P. Vavassori, The 2022 magneto-optics roadmap. *J. Phys. D Appl. Phys.* **55**, 463003 (2022).

29. J. McCord, Progress in magnetic domain observation by advanced magneto-optical microscopy. *J. Phys. D Appl. Phys.* **48**, 333001 (2015).

30. S. Kumar, S. Kumar, Ultrafast terahertz spin and orbital transport in magnetic/nonmagnetic multilayer heterostructures and a perspective. *J. Appl. Phys.* **134**, 170901 (2023).

31. J. He, S. Li, T. Frauenheim, Z. Zhou, Ultrafast laser pulse induced transient ferrimagnetic state and spin relaxation dynamics in two-dimensional antiferromagnets. *Nano Lett.* **23**, 8348–8354 (2023).

32. C. A. Ullrich, *Time-Dependent Density-Functional Theory: Concepts and Applications* (Oxford Univ. Press, 2011).

33. S. Meng, E. Kaxiras, Real-time, local basis-set implementation of time-dependent density functional theory for excited state dynamics simulations. *J. Chem. Phys.* **129**, 054110 (2008).

34. C. Lian, S.-J. Zhang, S.-Q. Hu, M.-X. Guan, S. Meng, Ultrafast charge ordering by self-amplified exciton-phonon dynamics in $TiSe_2$. *Nat. Commun.* **11**, 43 (2020).

35. Q. Cai, Y. Zhang, D. Luong, C. A. Tulk, B. P. Fokwa, C. Li, Spin-phonon interactions and anharmonic lattice dynamics in Fe_3GeTe_2. *Adv. Phys. Res.* , 2200089 (2023).

36. J. Wu, Y. Yao, M.-L. Lin, M. Rösner, Z. Du, K. Watanabe, T. Taniguchi, P.-H. Tan, S. Haas, H. Wang, Spin-phonon coupling in ferromagnetic monolayer chromium tribromide. *Adv. Mater.* **34**, 2108506 (2022).

37. K. Kim, J. Seo, E. Lee, K.-T. Ko, B. S. Kim, B. G. Jang, J. M. Ok, J. Lee, Y. J. Jo, W. Kang, J. H. Shim, C. Kim, H. W. Yeom, B. Il Min, B.-J. Yang, J. S. Kim, Large anomalous Hall current induced by topological nodal lines in a ferromagnetic van der Waals semimetal. *Nat. Mater.* **17**, 794–799 (2018).

38. M. Zhao, B.-B. Chen, Y. Xi, Y. Zhao, H. Xu, H. Zhang, N. Cheng, H. Feng, J. Zhuang, F. Pan, X. Xu, W. Hao, W. Li, S. Zhou, S. X. Dou, Y. Du, Kondo holes in the two-dimensional itinerant ising ferromagnet Fe_3GeTe_2. *Nano Lett.* **21**, 6117–6123 (2021).

39. Y. Deng, Y. Yu, Y. Song, J. Zhang, N. Z. Wang, Z. Sun, Y. Yi, Y. Z. Wu, S. Wu, J. Zhu, J. Wang, X. H. Chen, Y. Zhang, Gate-tunable room-temperature ferromagnetism in two-dimensional Fe_3GeTe_2. *Nature* **563**, 94–99 (2018).

40. H. L. Zhuang, P. R. C. Kent, R. G. Hennig, Strong anisotropy and magnetostriction in the two-dimensional Stoner ferromagnet Fe_3GeTe_2. *Phys. Rev. B* **93**, 134407 (2016).

41. J. Guo, C. Zhang, W. Liang, X.-X. Zhang, S. N. Luo, Enhanced coherent phonon excitation in Fe_3GeTe_2 via resonance Raman effect. *Phys. Rev. B* **103**, 024302 (2021).

42. C. Brennan, A. G. Joly, C.-F. Wang, T. Xie, B. T. O'Callahan, K. Crampton, A. Teklu, L. Shi, M. Hu, Q. Zhang, N. Kuthirummal, H. S. Arachchige, A. Chaturvedi, H. Zhang, D. Mandrus, C. Gong, Y. Gong, Strong surface-enhanced coherent phonon generation in van der Waals materials. *J. Phys. Chem. Lett.* **15**, 10442–10450 (2024).

43. A. de la Torre, D. M. Kennes, M. Claassen, S. Gerber, J. W. McIver, M. A. Sentef, Colloquium: Nonthermal pathways to ultrafast control in quantum materials. *Rev. Mod. Phys.* **93**, 041002 (2021).

44. D. Xiao, M.-C. Chang, Q. Niu, Berry phase effects on electronic properties. *Rev. Mod. Phys.* **82**, 1959–2007 (2010).

45. M. Gradhand, D. V. Fedorov, F. Pientka, P. Zahn, I. Mertig, B. L. Györffy, First-principle calculations of the Berry curvature of Bloch states for charge and spin transport of electrons. *J. Phys. Condens. Matter* **24**, 213202 (2012).

46. T. M. McCormick, N. Trivedi, Tuning the Chern number and Berry curvature with spin-orbit coupling and magnetic textures. *Phys. Rev. A* **91**, 063609 (2015).

47. J. Zhang, X. Tan, M. Liu, S. W. Teitelbaum, K. W. Post, F. Jin, K. A. Nelson, D. N. Basov, W. Wu, R. D. Averitt, Cooperative photoinduced metastable phase control in strained manganite films. *Nat. Mater.* **15**, 956–960 (2016).

48. A. Hubert, R. Schäfer, *Magnetic Domains: The Analysis of Magnetic Microstructures* (Springer Science & Business Media, 1998).

49. C. Wang, D. Chen, Y. Wang, S. Meng, Directional pumping of coherent phonons and quasiparticle renormalization in a dirac nodal-line semimetal. *Phys. Rev. X* **15**, 021053 (2025).

50. M. Che, W. Chen, M. Wang, F. M. Bartram, L. Liu, X. Dong, J. Liu, Y. Li, H. Lin, Z. Wang, E. Liu, Y. Yao, Z. Yuan, G.-M. Zhang, L. Yang, Discovery of terahertz-frequency orbitally coupled magnons in a Kagome ferromagnet. *Sci. Adv.* **11**, eadw1182 (2025).

51. Z. Wang, T. Sun, Z. Jiang, M. Yuan, Y. Huang, Y. Ren, D. Hou, T. Li, X. Liu, X. Luo, Y. Chai, A. Kimel, Y. Sun, Z. Sheng, Acceleration of ultrafast demagnetization in van der Waals ferromagnet Fe_3GeTe_2 in high magnetic field. *Natl. Sci. Rev.* **12**, nwaf185 (2025).

52. H. Probst, C. Möller, M. Schumacher, T. Brede, J. K. Dewhurst, M. Reutzel, D. Steil, S. Sharma, G. S. M. Jansen, S. Mathias, Unraveling femtosecond spin and charge dynamics with extreme ultraviolet transverse MOKE spectroscopy. *Phys. Rev. Res.* **6**, 013107 (2024).

53. C. Möller, H. Probst, J. Otto, K. Stroh, C. Mahn, S. Steil, V. Moshnyaga, G. S. M. Jansen, D. Steil, S. Mathias, Ultrafast element-resolved magneto-optics using a fiber-laser-driven extreme ultraviolet light source. *Rev. Sci. Instrum.* **92**, 065107 (2021).

54. N. Nagaosa, J. Sinova, S. Onoda, A. H. MacDonald, N. P. Ong, Anomalous Hall effect. *Rev. Mod. Phys.* **82**, 1539–1592 (2010).

55. T. Jungwirth, Q. Niu, A. H. MacDonald, Anomalous Hall effect in ferromagnetic semiconductors. *Phys. Rev. Lett.* **88**, 207208 (2002).

56. M. Schüler, U. D. Giovannini, H. Hübener, A. Rubio, M. A. Sentef, P. Werner, Local Berry curvature signatures in dichroic angle-resolved photoelectron spectroscopy from two-dimensional materials. *Sci. Adv.* **6**, eaay2730 (2020).

57. J. W. McIver, B. Schulte, F.-U. Stein, T. Matsuyama, G. Jotzu, G. Meier, A. Cavalleri, Light-induced anomalous Hall effect in graphene. *Nat. Phys.* **16**, 38–41 (2019).

58. C. Lian, M. Guan, S. Hu, J. Zhang, S. Meng, Photoexcitation in solids: First-principles quantum simulations by real-time TDDFT. *Adv. Theory Simul.* **1**, 1800055 (2018).

59. P. Giannozzi, S. Baroni, N. Bonini, M. Calandra, R. Car, C. Cavazzoni, D. Ceresoli, G. L. Chiarotti, M. Cococcioni, I. Dabo, A. Dal Corso, S. De Gironcoli, S. Fabris, G. Fratesi, R. Gebauer, U. Gerstmann, C. Gougoussis, A. Kokalj, M. Lazzeri, L. Martin-Samos, N. Marzari, F. Mauri, R. Mazzarello, S. Paolini, A. Pasquarello, L. Paulatto, C. Sbraccia, S. Scandolo, G. Sclauzero, A. P. Seitsonen, A. Smogunov, P. Umari, R. M. Wentzcovitch, QUANTUM ESPRESSO: A modular and open-source software project for quantum simulations of materials. *J. Phys. Condens. Matter* **21**, 395502 (2009).

60. P. Giannozzi, O. Andreussi, T. Brumme, O. Bunau, M. Buongiorno Nardelli, M. Calandra, R. Car, C. Cavazzoni, D. Ceresoli, M. Cococcioni, N. Colonna, I. Carnimeo, A. Dal Corso, S. de Gironcoli, P. Delugas, R. A. DiStasio, A. Ferretti, A. Floris, G. Fratesi, G. Fugallo, R. Gebauer, U. Gerstmann, F. Giustino, T. Gorni, J. Jia, M. Kawamura, H.-Y. Ko, A. Kokalj, E. Küçükbenli, M. Lazzeri, M. Marsili, N. Marzari, F. Mauri, N. L. Nguyen, H.-V. Nguyen, A. Otero-de-la Roza, L. Paulatto, S. Poncé, D. Rocca, R. Sabatini, B. Santra, M. Schlipf, A. P. Seitsonen, A. Smogunov, I. Timrov, T. Thonhauser, P. Umari, N. Vast, X. Wu, S. Baroni, Advanced capabilities for materials modelling with Quantum ESPRESSO. *J. Phys. Condens. Matter* **29**, 465901 (2017).

61. P. Giannozzi, O. Baseggio, P. Bonfà, D. Brunato, R. Car, I. Carnimeo, C. Cavazzoni, S. De Gironcoli, P. Delugas, F. Ferrari Ruffino, A. Ferretti, N. Marzari, I. Timrov, A. Urru, S. Baroni, Quantum ESPRESSO toward the exascale. *J. Chem. Phys.* **152**, 154105 (2020).

62. M. A. Marques, N. T. Maitra, F. M. Nogueira, E. K. Gross, A. Rubio, *Fundamentals of Time-Dependent Density Functional Theory*, vol. 837 (Springer, 2012).

63. E. Runge, E. K. U. Gross, Density-functional theory for time-dependent systems. *Phys. Rev. Lett.* **52**, 997–1000 (1984).

64. Z. Wang, S.-S. Li, L.-W. Wang, Efficient real-time time-dependent density functional theory method and its application to a collision of an ion with a 2D material. *Phys. Rev. Lett.* **114**, 063004 (2015).

65. J. C. Tully, Mixed quantum-classical dynamics. *Faraday Discuss.* **110**, 407–419 (1998).

66. S. Baroni, S. de Gironcoli, A. Dal Corso, P. Giannozzi, Phonons and related crystal properties from density-functional perturbation theory. *Rev. Mod. Phys.* **73**, 515–562 (2001).

67. J. P. Perdew, A. Zunger, Self-interaction correction to density-functional approximations for many-electron systems. *Phys. Rev. B* **23**, 5048–5079 (1981).

68. W. Kohn, L. J. Sham, Self-consistent equations including exchange and correlation effects. *Phys. Rev.* **140**, A1133–A1138 (1965).

69. D. R. Hamann, M. Schlüter, C. Chiang, Norm-conserving pseudopotentials. *Phys. Rev. Lett.* **43**, 1494–1497 (1979).

70. D. R. Hamann, Optimized norm-conserving Vanderbilt pseudopotentials. *Phys. Rev. B* **88**, 085117 (2013).

71. M. J. van Setten, M. Giantomassi, E. Bousquet, M. J. Verstraete, D. R. Hamann, X. Gonze, G.-M. Rignanese, The PseudoDojo: Training and grading a 85 element optimized norm-conserving pseudopotential table. *Comput. Phys. Commun.* **226**, 39–54 (2018).

72. H. J. Monkhorst, J. D. Pack, Special points for Brillouin-zone integrations. *Phys. Rev. B* **13**, 5188–5192 (1976).

73. H.-J. Deiseroth, K. Aleksandrov, C. Reiner, L. Kienle, R. K. Kremer, Fe_3GeTe_2 and Ni_3GeTe_2—Two new layered transition-metal compounds: Crystal structures, HRTEM investigations, and magnetic and electrical properties. *Eur. J. Inorg. Chem.* **2006**, 1561–1567 (2006).

74. G. Pizzi, V. Vitale, R. Arita, S. Blügel, F. Freimuth, G. Géranton, M. Gibertini, D. Gresch, C. Johnson, T. Koretsune, J. Ibañez-Azpiroz, H. Lee, J.-M. Lihm, D. Marchand, A. Marrazzo, Y. Mokrousov, J. I. Mustafa, Y. Nohara, Y. Nomura, L. Paulatto, S. Poncé, T. Ponweiser, J. Qiao, F. Thöle, S. S. Tsirkin, M. Wierzbowska, N. Marzari, D. Vanderbilt, I. Souza, A. A. Mostofi, J. R. Yates, Wannier90 as a community code: New features and applications. *J. Phys. Condens. Matter* **32**, 165902 (2020).

75. Q. Wu, S. Zhang, H.-F. Song, M. Troyer, A. A. Soluyanov, WannierTools: An open-source software package for novel topological materials. *Comput. Phys. Commun.* **224**, 405–416 (2018).

76. E. Anastassakis, E. Burstein, A. A. Maradudin, R. Minnick, Morphic Effects-IV. Effects of an applied magnetic field on first-order photonoptical phonon interactions in non-magnetic crystals. *J. Phys. Chem. Solid* **33**, 1091–1103 (1972).
